# Dose-Dependent Response to Infection with Ebola Virus in the Ferret Model and Evidence of Viral Evolution in the Eye

**DOI:** 10.1128/JVI.00833-21

**Published:** 2021-11-23

**Authors:** Robert J. Watson, Julia Tree, Susan A. Fotheringham, Yper Hall, Xiaofeng Dong, Kimberley Steeds, Jade Gouriet, Francisco J. Salguero, Christopher Burton, James Pitman, Linda Easterbrook, Kevin S. Richards, Jane Burton, Kevin Bewley, Christine Bruce, Julian A. Hiscox, Miles W. Carroll, Simon G. P. Funnell

**Affiliations:** a UK Health Security Agency, Porton Down, Salisbury, United Kingdom; b Institute of Infection, Veterinary and Ecological Sciences, University of Liverpool, Liverpool, United Kingdom; c A*STAR Infectious Diseases Laboratories (A*STAR ID Labs), Agency for Science, Technology and Research (A*STAR), Singapore; d Nuffield Department of Medicine, Oxford University, Oxford, United Kingdom; University of Kentucky College of Medicine

**Keywords:** ferret, Ebola, filovirus, animal model, eye, BARDA, EBOV, immune privileged, PHE, Porton Down, medical countermeasure, UKHSA

## Abstract

Filoviruses cause high-consequence infections with limited approved medical countermeasures (MCMs). MCM development is dependent upon well-characterized animal models for the assessment of antiviral agents and vaccines. Following large-scale Ebola virus (EBOV) disease outbreaks in Africa, some survivors are left with long-term sequelae and persistent virus in immune-privileged sites for many years. We report the characterization of the ferret as a model for Ebola virus infection, reproducing disease and lethality observed in humans. The onset of clinical signs is rapid, and EBOV is detected in the blood, oral, and rectal swabs and all tissues studied. We identify viral RNA in the eye (a site of immune privilege) and report on specific genomic changes in EBOV present in this structure. Thus, the ferret model has utility in testing MCMs that prevent or treat long-term EBOV persistence in immune-privileged sites.

**IMPORTANCE** Recent reemergence of Ebola in Guinea that caused over 28,000 cases between 2013 and 2016 has been linked to the original virus from that region. It appears the virus has remained in the region for at least 5 years and is likely to have been maintained in humans. Persistence of Ebola in areas of the body for extended periods of time has been observed, such as in the eye and semen. Despite the importance of reintroduction of Ebola from this route, such events are rare in the population, which makes studying medical interventions to clear persistent virus difficult. We studied various doses of Ebola in ferrets and detected virus in the eyes of most ferrets. We believe this model will enable the study of medical interventions that promote clearance of Ebola virus from sites that promote persistence.

## INTRODUCTION

Ebola virus disease (EVD) is a severe human viral hemorrhagic fever caused by infection with the filoviruses, including Bundibugyo virus (BDV), Ebola virus (EBOV), Sudan virus (SUDV), and Tai Forest virus (TAFV) (1). The viruses that cause EVD are located mainly in sub-Saharan Africa. People can get EVD through direct contact with an infected animal (bat or nonhuman primate) or a sick or dead person infected with Ebola virus (2). Signs and symptoms typically start between 2 days and 3 weeks after contracting the virus. These include a fever, sore throat, muscular pain, and headaches. Vomiting, diarrhea, and rash usually follow, along with decreased function of the liver and kidneys. Gastrointestinal symptoms and significant fluid loss can lead to hypovolemic shock and sudden death. Hemorrhagic symptoms can also occur but are not a defining feature.

From February to June 2021 there was an outbreak of EVD in Guinea, West Africa, where 16 cases were confirmed and 12 people died (3). The genomic sequence data imply this outbreak is from the reintroduction of the virus strain that caused the 2014–2016 EBOV outbreak in this region, which remains the largest recorded outbreak in history (4). This large outbreak has led to a greater understanding of the pathogenesis of EBOV (5), has provided the first opportunity to test and develop medical countermeasures (MCMs) against filoviruses, and has provided clear and compelling evidence that EBOV can persist in survivors, likely in immune-privileged sites for >500 days following infection, and can lead to persistent arthralgia, arthritis, and hearing loss (6, 7). At least half of survivors surveyed following the 2014–2016 outbreak complained of ocular complications such as blurry vision and discomfort (8), and one-fifth of these survivors were diagnosed with uveitis. The effect of EVD on the eye has stimulated research in this area, both *in vitro* (9) and *in vivo* (10–14), and vision problems have been noted 3 years after initial infection (15), although the number of studies performed is still limited. Genomic copies of EBOV have been detected in semen in about 10% of survivors more than 1 year after infection (16), which represents a large potential reservoir for reinfection through sexual activity and is thought to be the cause of the recent outbreak. In the limited number of humans studied, EBOV persistence has been associated with very few changes in genome sequence (17).

Animal models are frequently used as a tool for the identification and development of MCMs against rare or particularly severe diseases that cannot be ethically studied through clinical trials or challenge studies. The utility of these animal models is only realized when effective treatment in the disease model is predictive of clinical benefit in humans; confidence in that condition requires detailed pathogenesis studies and natural history studies to ensure the human disease resembles that in the animal model and mechanism of action of the medical countermeasure is translatable to human disease. These models are indispensable for the licensure of novel therapeutics through the FDA Animal Rule (FDA Animal Rule 2015), where the efficacy of a product is demonstrated by challenge studies in well-characterized animal models of the disease. To date, in EBOV infections, the nonhuman primate (NHP) model has been the primary model for characterizing MCMs and is considered the gold standard model used to test medical interventions (18). In fact, this model was used to identify and establish the proper regimen for the three products recently approved by the FDA for prophylaxis and treatment of EBOV (ERVEBO, INMAZEB, and EBANGA). Experiments using these animals, however, are laborious, expensive, and sometimes difficult to schedule, since the supply of suitable animals is limited, reinforcing the observation that an alternative smaller animal model would be attractive.

NHPs are susceptible to the filovirus strains that cause mortality and morbidity in humans and display many of the features of human disease, including uncontrolled systemic viral replication, vascular leakage, coagulopathy, and heightened immune responses (12, 19, 20). Thorough examination of nonhuman primate survivors of filovirus challenges has shown that a small percent display evidence for persistent virus in semen or in the eye, but the frequency of this observation and the cost and labor associated with nonhuman primate models makes the study of persistent virus in this model difficult (12, 21). The only small, nonprimate animal model susceptible to nonadapted strains of filovirus is the domestic ferret (Mustela putorius furo), which can be readily infected with wild-type virus. The ferret model has been described previously as a lethal small-animal model for EBOV (22), SUDV, BDV (23, 24), and Reston virus (25), although Marburg and Ravn viruses fail to cause disease in this species (26).

While others have described the course of EBOV infection in the ferret model before, in this work we further extend and characterize the model by investigating the effect of challenge dose. We were able to demonstrate that the ferret is extremely sensitive to EBOV infection with mortality observed in all animals that displayed symptoms and had detectable EBOV. Additionally, we were able to detect EBOV in the eye of most infected animals, suggesting that a ferret model can be developed to test treatments designed to reduce the persistent virus observed in humans. We also investigated the diversity arising after infection in the consensus viral genome sequences in different organs (lung, liver, spleen, kidney, reproductive tract, and eye) of Ebola-infected ferrets for the first time. We were able to identify minor variations in nucleic acid sequence of the viral genome in different tissues using RNA-sequencing (RNA-seq), and we also report on the histology of EBOV infection in the eye.

## RESULTS

### Study design.

Ferrets were challenged intramuscularly with a well-characterized EBOV-Kikwit-95 stock (see Fig. S1 and Table S1 in the supplemental material). EBOV in 100 μl phosphate-buffered saline (PBS) was injected into the right cranial thigh muscle at six different doses from 5.6 × 10^4^ to 5.6 × 10^−3^ 50% tissue culture infectious dose (TCID_50_) over four studies. Following challenge, blood, oral, and rectal swabs were sampled in a staggered daily scheme (Fig. S2). The ferrets were monitored twice daily until clinical signs were first noted. Frequency of monitoring was then increased to 4 to 6 times per 24-h period when at least one clinical sign was consistently observed (more than two consecutive time points). Each day at least one ferret from each group was sampled. Blood from the vena cava was split into lithium heparin for blood chemistry and EDTA for PCR. Oral and rectal swabs were placed into 1 ml viral transport medium for viral quantification. One animal from the first study was removed from all analyses due to a procedural issue encountered during challenge; all of the subsequent samples taken were negative for Zaire ebolavirus RNA and the ferret remained healthy.

### Challenge dose of EBOV correlates with survival time.

**FIG 1:**
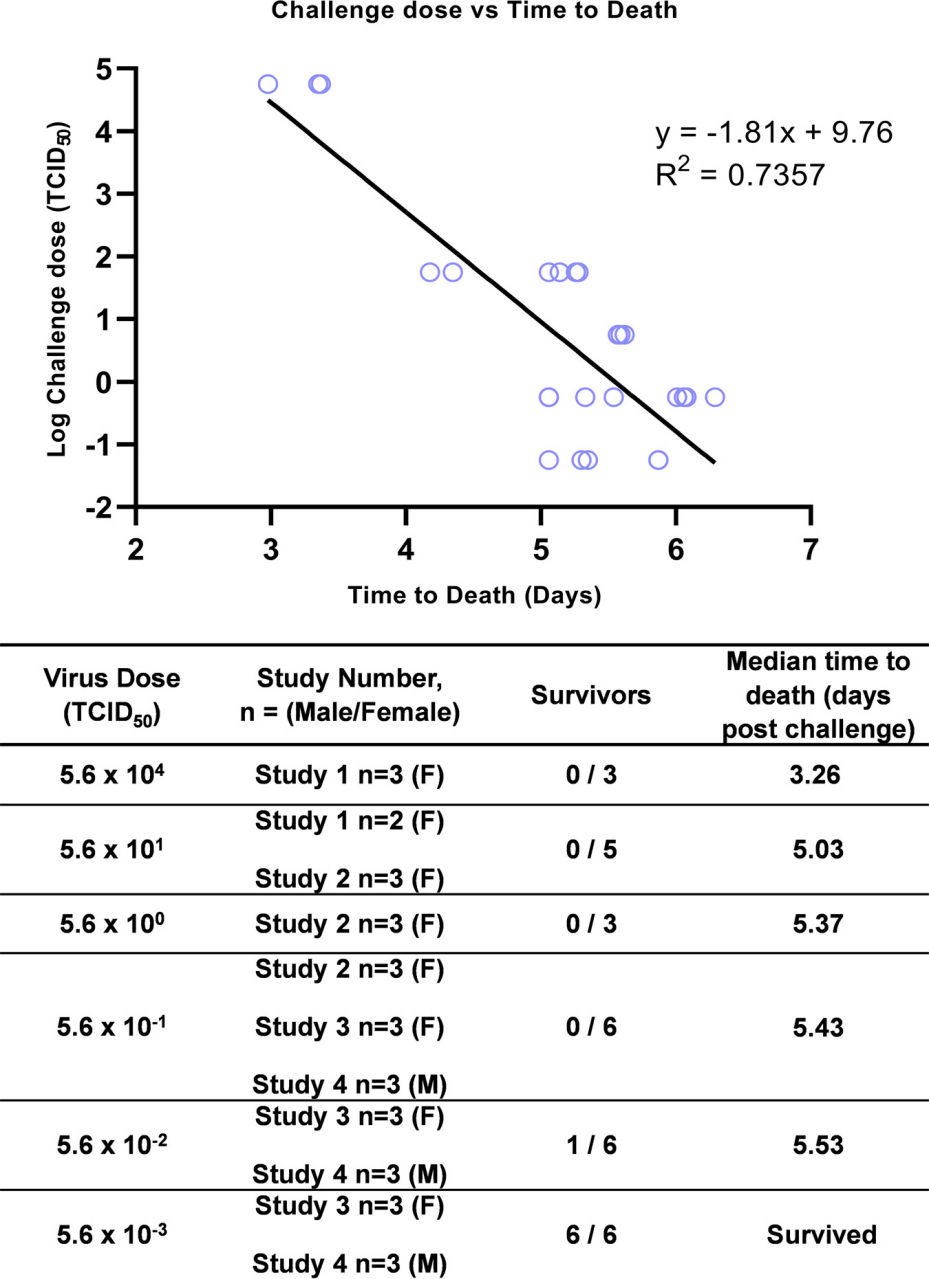
Dose survival. Regression of all survival times versus log challenge dose. Median time to death in days for each challenge dose and number of ferrets at each dose (*n*).

All 20 ferrets challenged with 5.6 × 10^−1^ TCID_50_ EBOV-Kikwit-95 or greater were euthanized within 7 days, as they all met the predetermined clinical signs qualifying for humane euthanasia. Five of 6 ferrets infected with 5.6 × 10^−2^ TCID_50_ were also euthanized within 7 days. The median survival of the 5.6 × 10^4^ TCID_50_ group was 3.26 days, the 5.6 × 10^1^ TCID_5_ group was 5.03 days, the 5.6 × 10^0^ TCID_50_ group was 5.37 days, the 5.6 × 10^−1^ TCID_50_ group was 5.53 days, and the 5.6 × 10^−02^ TCID_50_ group (5/6) was 5.53 days. All ferrets challenged with 5.6 × 10^−02^ TCID_50_ survived to the end of the 14-day study period. Median time to death was closely correlated with the challenge dose (Fig. 1).

### Clinical signs rise rapidly shortly before euthanasia.

**FIG 2:**
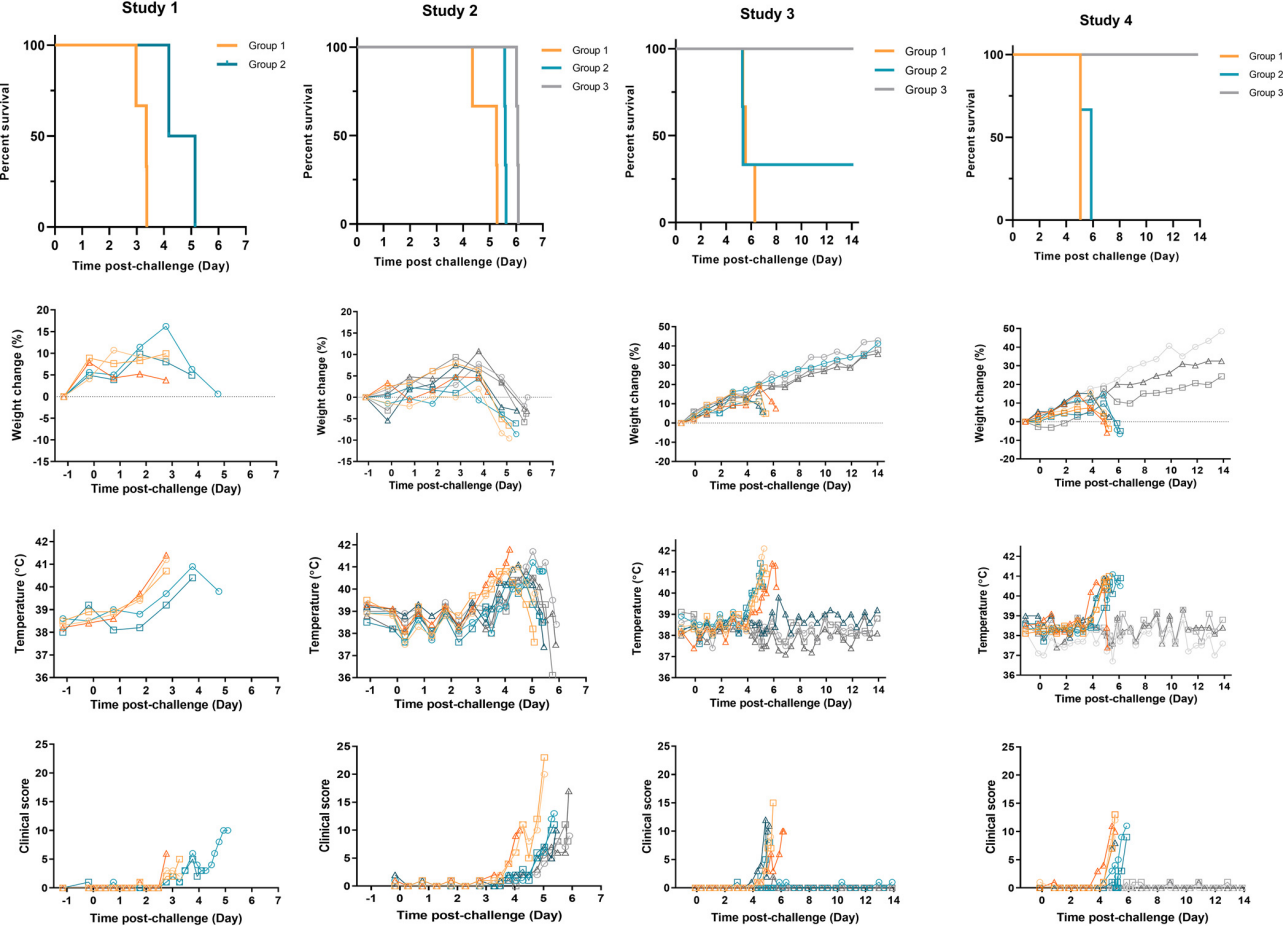
Survival and clinical observations. (a) Survival of ferrets was plotted as a Kaplan-Meier survival curve calculated by postchallenge time to euthanasia decision. (b) Weight was recorded daily, and percent weight change from baseline was plotted. Points show values for individual animals. (c) Temperatures were taken at the same time as clinical observations, using the identifier chip, to ensure any peak of fever was recorded. Individual animal temperatures are displayed on the graph. (d) Clinical observations were carried out twice daily four to six times daily during the period of onset of clinical symptoms. Observations were summed for each ferret based the following scores: healthy, 0; arched back, 1; dehydrated/not drinking, 1; rash, 1; gait changes, 1; wasp waisted, 1; ruffled fur, 1; nasal discharge, 1; sneezing, 1; shivering, 1; lethargic, 2; vomiting, 2; depression, 2; diarrhea, 2; labored breathing, 3; temperature, >39°C, 1; temperature, >40°C, 2; weight loss, >5% from maximum, 1; weight loss, >10% from maximum, 2. *n* = 3 ferrets per group. Study 1, group 1, 5.6 × 10^4^ TCID_50_, orange lines; group 2, 5.6 × 10^1^ TCID_50_ teal lines; study 2, group 1, 5.6 × 10^1^ TCID_50_, orange lines; group 2, 5.6 × 10^0^ TCID_50_ teal lines; group 3, 5.6 × 10^−1^ TCID_50_ gray lines. Study 3, group 1, 5.6 × 10^−1^ TCID_50_, orange lines; group 2, 5.6 × 10^−2^ TCID_50_ teal lines; group 3, 5.6 × 10^−3^ TCID_50_ gray lines. Study 4 group 1, 5.6 × 10^−1^ TCID_50_, orange lines; group 2, 5.6 × 10^−2^ TCID_50_ teal lines; group 3, 5.6 × 10^−3^ TCID_50_ gray lines.

The total of clinical scores for each group of ferrets is shown in Fig. 2d, and total scores at euthanasia decision are shown in Table S2. In the first study, 5/5 ferrets in the high- and low-dose groups showed multiple clinical signs of disease, including gait changes and arched back. The mean summed clinical score for this study was 5. With the lower doses of challenge given in the second study, the ferrets took longer to reach the euthanasia endpoint compared to the first study, and all of ferrets displayed multiple clinical signs with a mean summed clinical score of 13. The third and fourth studies produced clinical scores similar to the first two studies. The most common clinical sign over all 4 studies was arched back, which was seen in 92% of all infected animals. Lethargy, gait change, and wasp waisted were the next most common signs and were observed in 80% of infected animals. Ruffled fur was seen in 72% of infected animals, a sign that these ferrets were not grooming regularly. Over half of the infected ferrets (60%) displayed labored breathing at euthanasia. Less common signs included dehydration (36%), sneezing, vomiting, and disorientation (all 8%). In rare cases (4%), shivering, diarrhea, depression, nasal discharge, and immobility were observed. After disease became obvious, progression to clinical scores warranting euthanasia was generally rapid, and in no case did this extend beyond 48 h and was typically 24 h (Fig. 2d). No significant clinical signs (maximum score, 2) were seen in any of the lowest-dose group, 5.6 × 10^−3^ TCID_50_ ferrets, which all survived until the end of the study.

Fever (>40°C) was observed in all of the ferrets that progressed to euthanasia, with the onset of fever observed approximately 24 h prior to clinical scores sufficient for euthanasia. The onset of fever (temperature, >39.5°C) was inversely proportional to the challenge dose, with temperatures above 40°C seen in the 5.6 × 10^4^ TCID_50_ dose group 24 h more rapidly than the 5.6 × 10^1^ TCID_50_ dose group. There was a 2-day difference of onset of fever between the highest and lowest lethal doses across all studies. No fever was observed in the lowest dose (5.6 × 10^−3^ TCID_50_) group (Fig. 2).

Weight loss was observed in 12 out of 32 ferrets (Fig. 2). None of these ferrets lost more than 10% of their initial body weight. Weight loss from maximum was also calculated to adjust for the initial increase in body weight. Using this calculation 23 out of 25 ferrets that were euthanized lost between 1.8% and 13.5% of their maximum body weight by euthanasia. In the 5.6 × 10^−3^ TCID_50_ low-dose group, all 6 animals increased in weight during the study. The magnitude of the weight loss was independent of the viral challenge dose.

### EBOV dissemination is widespread in ferrets.

Viral load was measured via RNA in blood, oral, and rectal swabs and tissues via reverse transcription-quantitative PCR (RT-qPCR). Viral RNA consistently appeared in blood from 2 days postchallenge and reached between 2 × 10^7^ genome copies/ml and 8 × 10^9^ genome copies/ml in blood for all ferrets that met their euthanasia endpoint. The final amount of RNA detected in each group was not dependent on the challenge dose of virus administered. Viral RNA levels in blood samples of all surviving animals were undetectable at any time up to the end of the 14-day study period.

**FIG 3:**
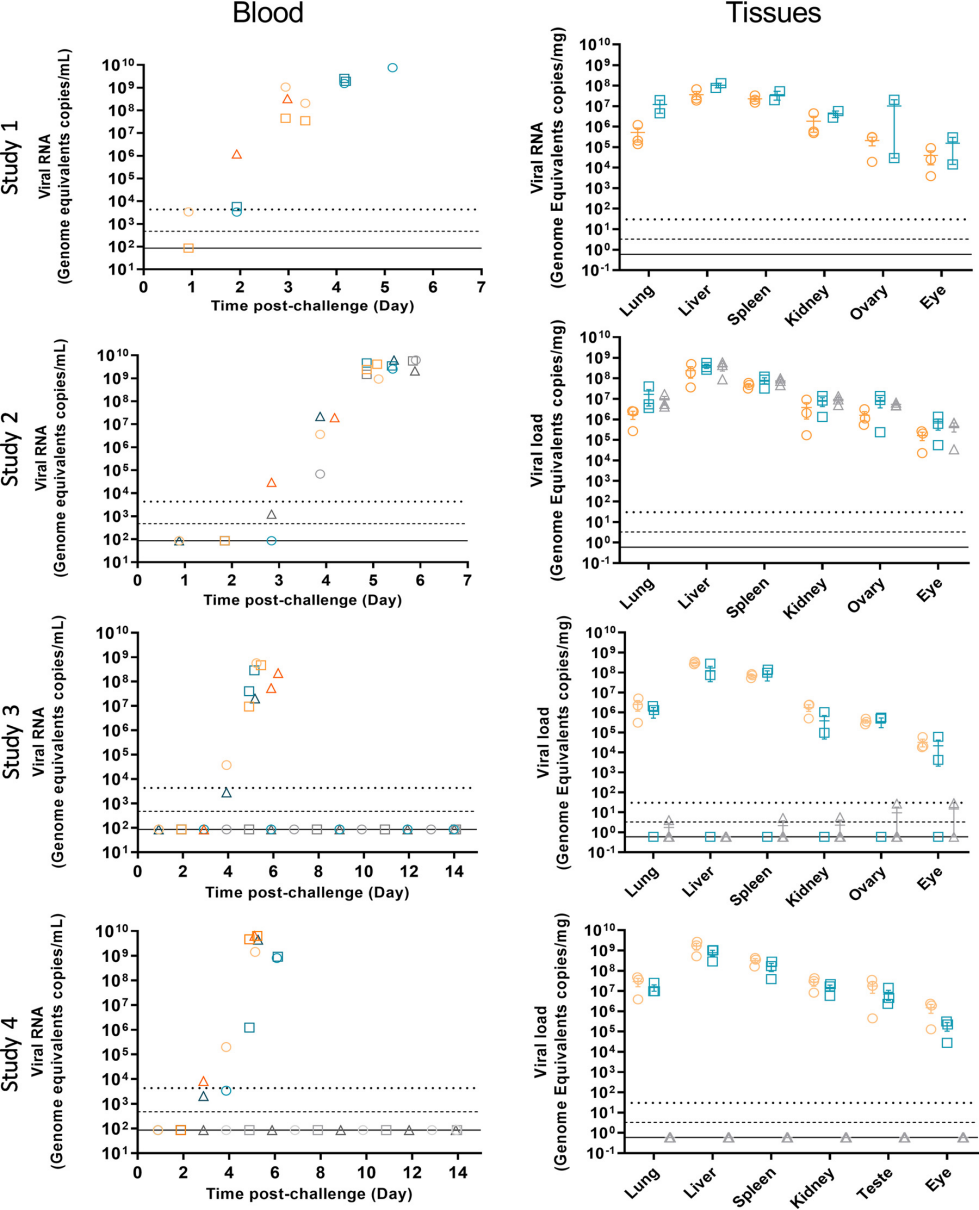
Viral load. Viral RNA was measured in the blood and tissues by RT-qPCR. Blood viral RNA was measured from one animal from each group at each time point. Tissue viral RNA was measured from each animal at euthanasia. The results are plotted as mean ± standard. Group 1, 5.6 × 10^−1^ TCID_50_, orange symbols; group 2, 5.6 × 10^−2^ TCID_50_, teal symbols; group 3, 5.6 × 10^−3^ TCID_50_, gray symbols. Upper dotted line is the lower limit of quantification (LLOQ). Dashed line is the limit of detection (LOD), and solid line is the theoretical minimum detectable amount (TDMA).

The highest levels of viral RNA in tissues were detected by RT-qPCR in the liver and spleen of ferrets euthanized before the end of the study (Fig. 3). Viral loads were lower in the lung and kidney and were lowest in the reproductive tract and eye. The magnitude of the EBOV genomic RNA at euthanasia was not dependent on the initial challenge dose, as all animals had comparable viral loads at euthanasia, implying the delayed time to euthanasia for lower challenge doses simply reflects the additional time required to reach the viral load commensurate with euthanasia. None of the animals that survived to the end of the study had detectable RNA levels in any tissue at the end of the study.

The timing and magnitude of the RNA detected in oral and rectal swabs was analogous to that observed from blood. Viral RNA in oral swab samples increased from day 3 to 4 and was at the highest level at the time of euthanasia for all animals (Fig. S3). Viral RNA in swab samples of the animals surviving to the end of the 14 day was not detected at any time.

**FIG 4:**
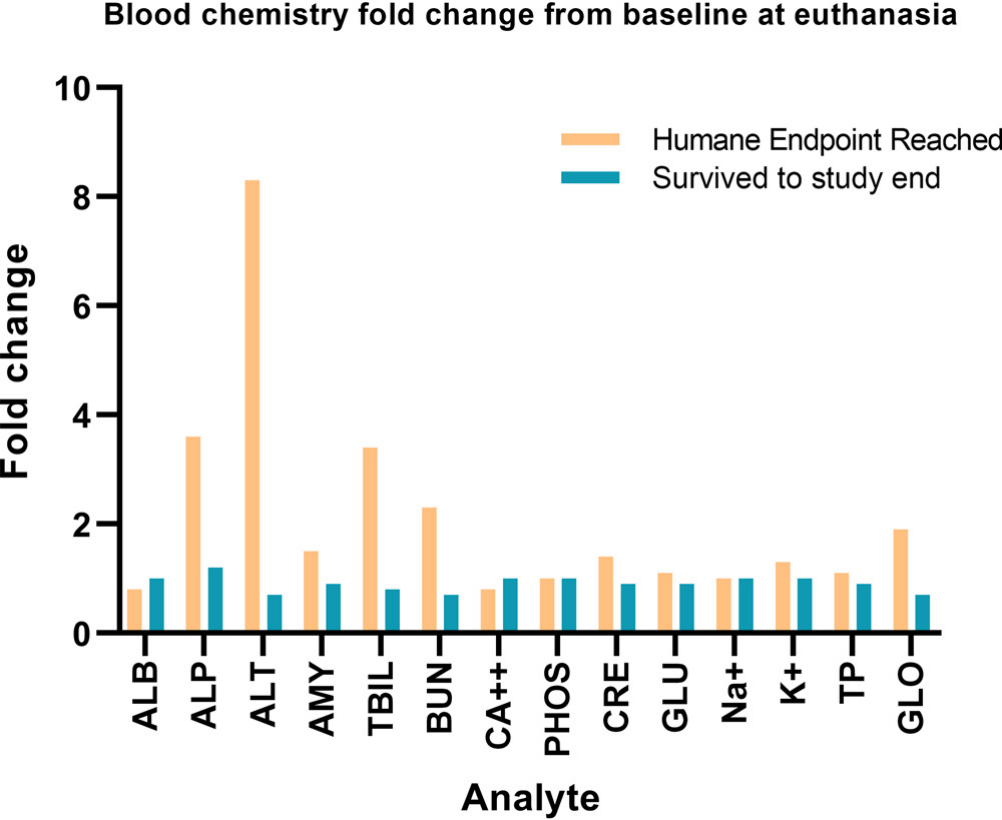
Blood chemistry fold change from baseline. The fold change from the mean baseline of all animals where humane endpoint was reached (orange bars; *n* = 25) and those animals that survived to the end of the study (teal bars; *n* = 6) with blood chemistry data available. The parameters measured were albumin (ALB), alanine aminotransferase (ALT), alkaline phosphatase (ALP), amylase (AMY), total bilirubin (TBIL), urea nitrogen (BUN), total calcium (CA^2+^), phosphorus (PHOS), creatinine (CRE), glucose (GLU), sodium (NA^+^), potassium (K^+^), globulin (GLOB), and total protein (TP) in heparinized whole blood.

Fourteen blood chemistry parameters were analyzed at each sampling day. The largest changes were seen at euthanasia endpoint (Fig. 4 and Fig. S4 and S5). Liver damage was indicated by elevated levels of alkaline phosphatase from baseline (3.6-fold), alanine aminotransferase (8.3-fold), total bilirubin (3.4-fold), and blood urea nitrogen (2.3-fold) in animals that progressed to euthanasia endpoint (Fig. 4). Animals were also becoming dehydrated, as indicated by increased levels of globulin (1.9-fold). For the animals that survived to the end of the study, none of the parameters was greater than 1.2-fold increased from baseline (Fig. 4).

### EBOV RNA is detected within immune-privileged sites in the absence of observable tissue damage.

**FIG 5:**
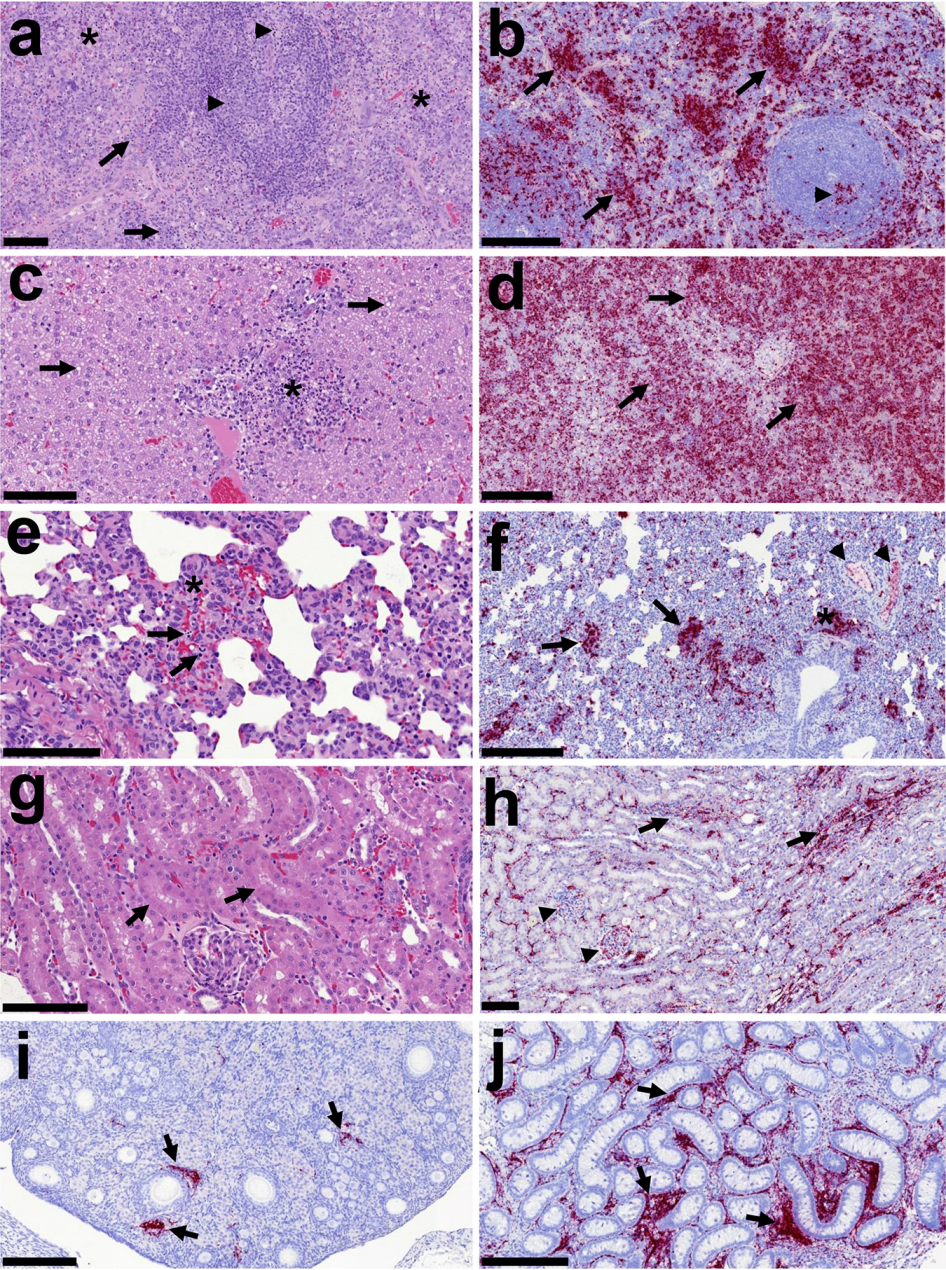
Histopathology and presence of virus RNA in tissues by *in situ* hybridization. (a) Spleen (study 2; 5.6 × 10^−1^ TCID_50_). Multifocal necrosis observed within the red pulp (*) with multiple degenerated and some viable PMNs. Lymphocyte cell death is observed within the red pulp (arrows) and the splenic follicles (arrowheads), with condensation and fragmentation of nuclear chromatin (necrosis or apoptosis). Bar, 100 μm. (b) Spleen (study 3; 5.6 × 10^−2^ TCID_50_). Virus RNA (RNASCope; red stain) within the red pulp (arrows) and splenic follicles (arrowhead). Bar, 200 μm. (c) Liver (study 2; 5.6 × 10^−0^ TCID_50_). Necrotic foci with abundant cell debris and inflammatory cell infiltration in a portal area are shown (*). Presence of moderate hepatocellular fat degeneration is shown (arrows). Bar, 100 μm. (d) Liver (study 3; 5.6 × 10^−1^ TCID_50_). Virus RNA (RNASCope; red stain) within the hepatic parenchyma is shown (arrows). Bar, 200 μm. (e) Lung (study 2; 5.6 × 10^−0^ TCID_50_). Interstitial pneumonia (*) with the presence of necrosis (arrows indicating cell death) and moderate congestion is shown. Bar, 100 μm. (f) Lung (study 4; 5.6 × 10^−1^ TCID_50_). Virus RNA (RNASCope; red stain) within areas of interstitial pneumonia (arrows), BALT (*), and blood vessels (arrowheads) are shown. Bar, 200 μm. (g) Kidney (study 4; 5.6 × 10^−2^ TCID_50_). Mild necrosis of tubular epithelial cells (arrows) and mild congestion. Bar, 100 μm. (h) Kidney (study 4; 5.6 × 10^−1^ TCID_50_). Virus RNA (RNASCope; red stain) within the cortical interstitium (arrows) and glomeruli (arrowheads) is shown. Bar, 100 μm. (i) Ovary (study 1; 5.6 × 10^4^ TCID_50_). Virus RNA (RNASCope; red stain) within the cortex (arrows) is shown. Bar, 200 μm. (j) Testis (study 4; 5.6 × 10^−1^ TCID_50_). Virus RNA (RNASCope; red stain) within the interstitium ovary is shown. Animal 122.19 is shown. Virus RNA (RNASCope, red stain) within the cortex (arrows) is shown. Bar, 200 μm.

The most severe lesions were observed in the spleen and the liver. The spleen showed severe multifocal necrosis within the red and white pulp, accompanied by moderate hemorrhaging and congestion. The necrotic foci were accompanied by the presence of abundant PMNs (viable and degenerated) (Fig. 5a). Mild splenic follicle lymphoid depletion was also observed (Fig. 5a). The liver showed moderate congestion and moderate to severe multifocal necrosis, together with hepatocellular fat degeneration and occasional inflammatory cell infiltrates (Fig. 5c). Occasionally, intracytoplasmic eosinophilic inclusion bodies were observed within the cytoplasm of hepatocytes. The lung showed very mild congestion/hemorrhage, associated with the presence of necrotic foci within the interalveolar septa and around bronchioles and bronchi (Fig. 5e). Kidney showed mild to moderate congestion and occasional mild tubular necrosis (Fig. 5g). Similarly, mild congestion was observed in the reproductive tract with no other lesion present in uterus, ovary, testes, or epididymis. No histopathological lesions were observed in the eye structures or the glands and appendages.

The presence of virus RNA in tissue sections by RNAScope is summarized in Table S3 (spleen, liver, kidney, lung, and reproductive tract) and Table S4 (eye). Virus RNA was observed in different cell types, mostly associated with the presence of histopathological lesions, but also highly associated with blood vessels in all the studied tissues, with positive staining within the endothelial lining and in circulating cells.

In the spleen, the largest amount of virus RNA was detected in the macrophages of the red pulp and the marginal zone and within the areas of necrosis (Fig. 5b). Within the splenic follicles occasional cells were stained positive for RNA. Where necrotic foci were affecting the white pulp, a larger number of cells and necrotic debris stained positive for RNA. In the liver, the presence of virus RNA was also associated with the presence of necrotic foci and inflammatory cell infiltration (Fig. 5d). Cells positive for EBOV RNA staining were mainly macrophages/Kupffer cells but also other inflammatory cells within the necrotic foci and portal areas. Hepatocytes were also positively stained mostly in the animals, showing high levels of virus RNA in the liver. In the lung, virus RNA was observed within the foci of necrosis and inflammation and in some scattered macrophages (Fig. 5f). In the kidney, virus RNA was observed in both the cortex and the medulla, mostly associated with blood vessels and inflammatory cell infiltrates (Fig. 5h). Virus RNA was detected in the glomeruli and occasionally in the epithelial tubular cells. In the reproductive tract, the virus RNA was present within the stroma of ovaries (Fig. 5i) and testis/epididymis (Fig. 5j) and associated with blood vessels in the oviduct.

**FIG 6:**
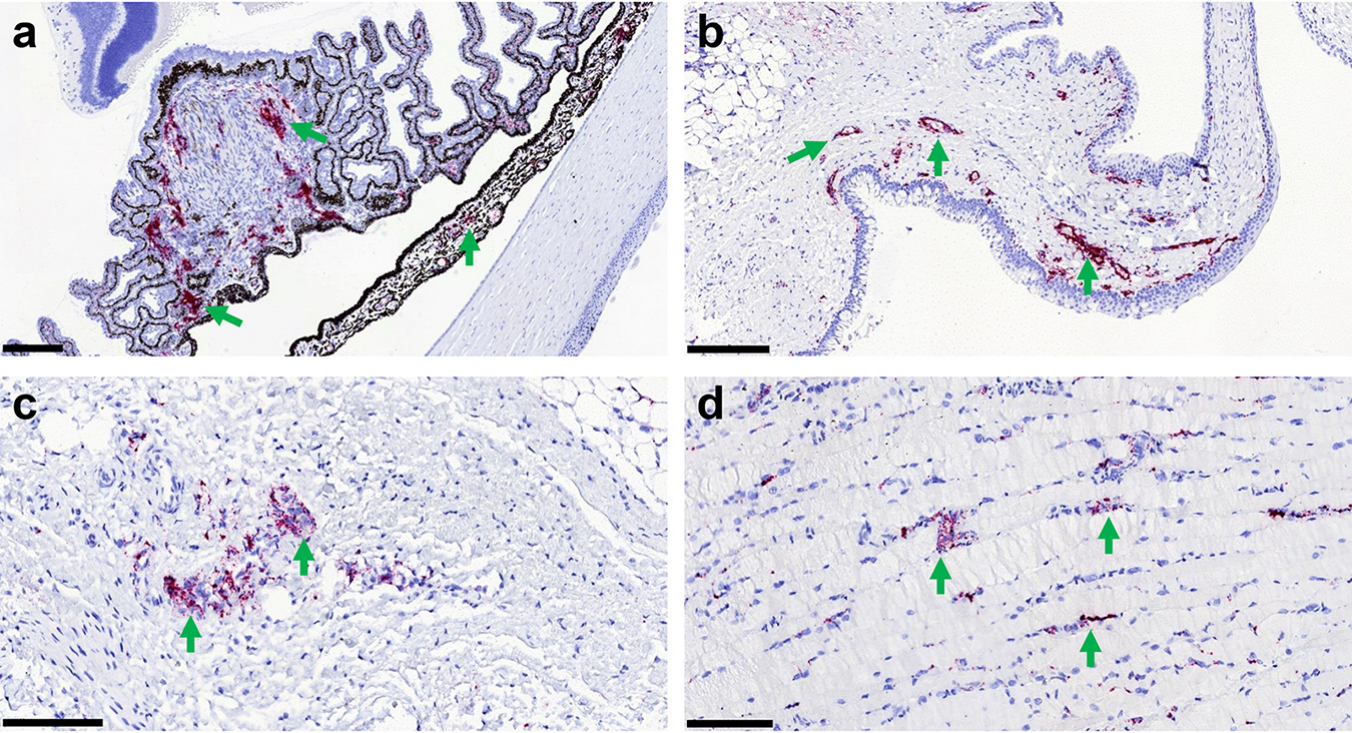
*In situ* hybridization of virus RNA in the eye structures. Detection of EBOV RNA by RNAScope ISH (red stain, arrows) in eye structures from experimentally inoculated ferrets. (a) Animal number 20016 (5.6 × 10^−1^ TCID_50_). Viral RNA within the iris and ciliary bodies is shown. Bar,  200 μm. (b) Animal number 01755 (5.6 × 10^−1^ TCID_50_). Viral RNA within the conjunctiva, mostly associated with blood vessels, is shown. Bar, 200 μm. (c) Animal number 20016 (5.6 × 10^−1^ TCID_50_). Viral RNA within the choroid is shown. Bar,  100 μm. (d) Animal number 01755 (5.6 × 10^−1^ TCID_50_). Viral RNA within the optic nerve is shown. Bar, 100 μm.

No histopathological lesions were found in the eyes. However, virus RNA was present in the eye from most of the infected animals (Table S4). The positive staining was mainly found associated with blood vessels from the choroid, iris, and ciliary bodies, together with the conjunctiva and adnexal glands (Fig. 6).

### Unique nucleotide changes are associated with EBOV in the eye and testes.

**FIG 7:**
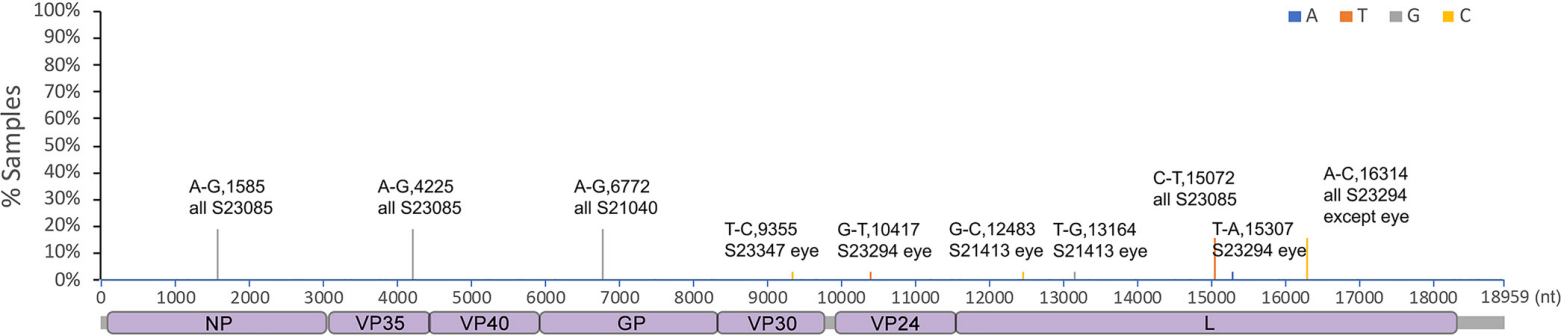
Sequencing analysis of virus RNA in the eye. Ebola virus was sequenced from the eyes, lung, liver, spleen, kidney, and ovaries of ferrets. Nucleotide mutations in the called consensus virus genomes were identified by comparison to the challenge virus genome sequence. Nucleotide change, position of the virus genome RNA, and the sample ID are shown on the top of the bar. Changes in the sequence that were specific to the eye are noted with eye at the end of the sample ID.

**FIG 8:**
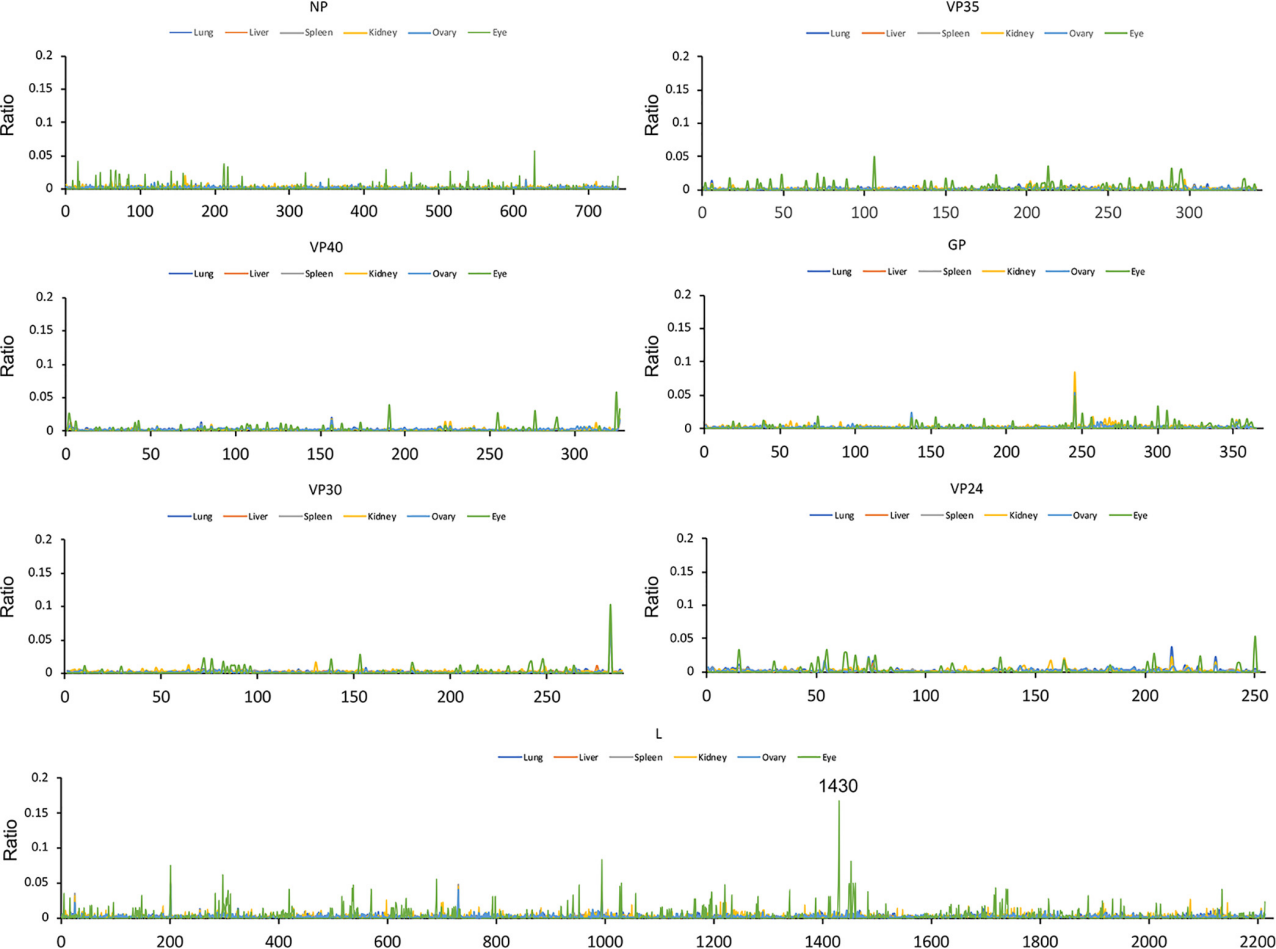
Minor variations in amino acids. Virus recovered from each tissue was analyzed for its variation compared to the challenge virus sequence. The ratio of variation was <50% in all organs. Only sites with amino acid coverage of  >5 were taken into account. The minor variation at L1430 showed an average variation ratio of 0.1667 (Q to stop) specifically in the eyes. Each tissue is represented by a different colored line. Lung, dark blue; liver, orange; spleen, gray; kidney, yellow; ovary, light blue; eye, green.

**FIG 9:**
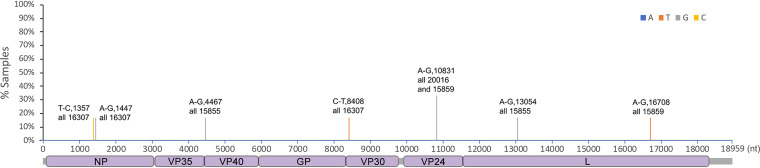
Sequencing analysis of virus RNA in the testes. Ebola virus was sequenced from the testes of ferrets. Nucleotide mutations in the called consensus virus genomes were identified by comparing to the challenge virus genome sequence. Nucleotide change, position of the virus genome RNA, and the sample ID are shown on the top of the bar.

**FIG 10:**
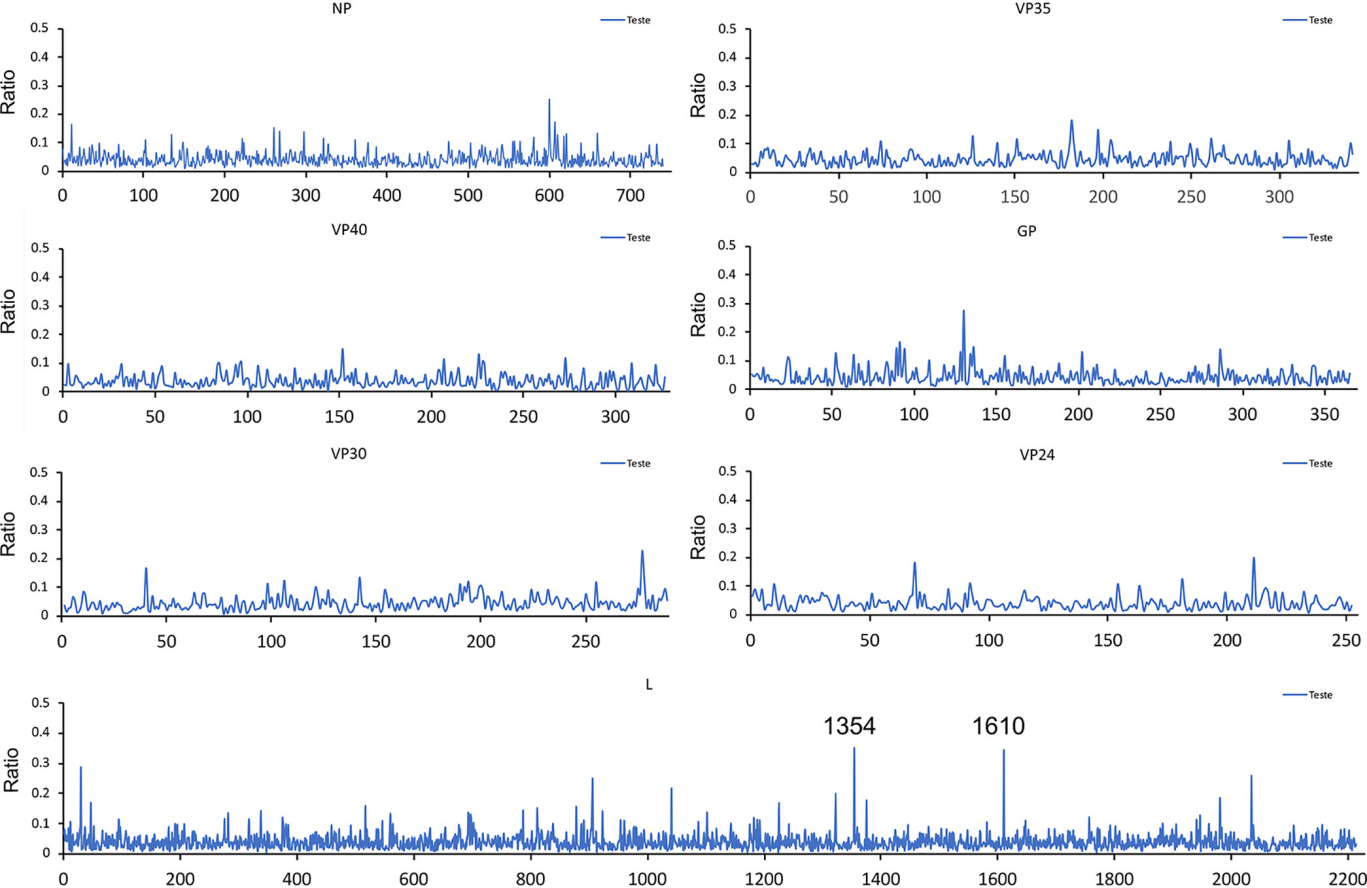
Minor variation of amino acids in the testes (ratio of variation <50%) in testes samples. Only sites with amino acid coverage of >5 were taken into account. Minor variation at L1354 and L1610 showed an average variation ratio of 0.3500 (majority, L to F) and 0.3447 (majority, R to Q), respectively.

The RNA sequences of EBOV genomes resident in different tissues were compared to investigate whether there was particular selection pressure associated with infection. For example, in the guinea pig model of EVD, specific mutations in VP24 are found that enhance pathogenesis (27), and in humans, variability and evolution of the initial viral sequence has been observed (28). The viral RNA sequence was determined and the consensus sequence compared between the lung, liver, spleen, kidney, ovary, and eyes (Fig. 7, Table 1). Consensus genomes and minor variants were detected, with the greatest genetic difference from the infecting virus observed in the genomic RNA detected in the eye (Fig. 8). One of the changes with the greatest frequency was the observation of a stop codon in approximately 16% of the genomes (Fig. 7). Differences of viral sequences in the testes were also observed using the ARTIC technique (Fig. 9, Table 2), and minor variants were detected (Fig. 10).

**TABLE 1:** Codon changes induced by the nucleotide mutation shown in Fig. 7

Genome position	Gene(s)	Nucleotide change	Reference codon	Reference amino acid	Codon change	Amino acid change	Sample ID
1585	NP	A-G	GAA	E	GAG	E	All S23085
4225	Noncoding, between VP35 and VP40	A-G					All S23085
6772	GP	A-G	GAA	E	GGA	G	All S21040
9355	VP30	T-C	TCT	S	CCT	P	S23347, eye only
10417	VP24	G-T	GAC	D	TAC	Y	S23294, eye only
12483	L	G-C	TTG	L	TTC	F	S21413, eye only
13164	L	T-G	ATT	I	ATG	M	S21413, eye only
15072	L	C-T	TTC	F	TTT	F	All S23085
15307	L	T-A	TTT	F	ATT	I	S23294, eye only
16314	L	A-C	GTA	V	GTC	V	S23294, eye only

**TABLE 2:** Codon changes induced by the nucleotide mutation shown in Fig. 9

Genome position	Gene	Nucleotide change	Reference codon	Reference amino acid	Codon change	Amino acid change	Sample ID
1375	NP	T-C	CTT	L	CTC	L	16307
1447	NP	A-G	GCA	A	GCG	A	16307
4467	Vp40	A-G	AAA	K	AAG	K	15855
8408	5′UTG VP30	C-T					16307
10831	VP24	A-G	AAA	K	GAA	E	20016 and 15859
13054	L	A-G	ACA	T	GCA	A	15855
16708	L	C-T	CAC	H	TAC	Y	15859

## DISCUSSION

Since the largest recorded EBOV outbreak in 2014 to 2016, which saw an estimated 11,323 deaths (https://www.who.int/news-room/fact-sheets/detail/ebola-virus-disease), there have been seven more outbreaks between 2017 and 2021 in the Democratic Republic of Congo (DRC) and Guinea, with deaths totaling 4,696. In the 5 years since the largest recorded outbreak, there have been 3 times the number of cases recorded for the prior 38 years. Two of these outbreaks are currently ongoing in North Kivu, DRC, with 2,287 deaths (https://reliefweb.int/report/democratic-republic-congo/democratic-republic-congo-drc-ebola-situation-report-39-march-12), and in Gouécké, Nzérékoré Region, Guinea, with 18 cases and 9 deaths. Although some vaccines and drugs are now approved by the FDA and becoming available for the prevention and treatment of EVD, there are still many unanswered questions about EVD and gaps in our treatment options that cannot be answered by awaiting fresh outbreaks. The clinical data from the large outbreak clearly demonstrated the persistence of EBOV in immune-privileged sites such as the testes and eyes. This population of EBOV carriers presents a unique public health threat, as recrudescence or transmission through sexual activity can provide a major source for reemergence of disease. The circulating virus in the latest outbreak in Guinea is startlingly similar to the Makona strain responsible for the 2014 to 2016 outbreak, suggesting the outbreak is the result of transmission from a latent carrier.

Animal models remain the most important tool for studying filovirus infections and for the identification and development of MCMs against the diseases caused by this class of viruses. In this work, we describe our studies to further investigate the potential for using the ferret as a small-animal model for MCM development, since it has been reported in the literature that it is susceptible to infection by authentic EBOV strains that can infect humans. In these studies, we demonstrated that the ferret is extremely susceptible to EBOV infection, consistent with the previously published studies of the ferret model of EBOV infection (23–25). In these dose-ranging studies, a systemic infection warranting euthanasia was observed at all doses of the challenge virus that could induce an infection. In the characterization of the viral infection, we observed that the infection was detectable in many organs, with slightly different RNA sequences observed in each organ. This diversity implies the animal viral load and disease pathogenesis are the result of a multifocal infection where the selective pressure at each organ results in a unique collection of sequences rather than replication at a single site followed by viral dissemination later in infection. In addition to finding evidence for viral replication in many tissues, EBOV RNA was also detected in the eyes and reproductive tracts of ferrets.

These studies characterizing the dose response to an intramuscular challenge of EBOV in ferrets is the first demonstration of localization of EBOV RNA in the eyes and reproductive tract of infected ferrets. These studies are preliminary, as we do not know if the ferrets will respond to MCMs after infection in a manner predictable for clinical benefit. It also is important to determine if the detection of EBOV in the eye at 76% will be reproducible in the context of MCM treatment or if the frequency in survivors will mirror the results seen in NHPs where EBOV was detectable in only about 10% of survivors’ eyes (12). Being able to detect virus consistently in the eye and reproductive tract of a small-animal model would provide a valuable tool to evaluate MCM effect on EBOV persistence in immune-privileged sites.

The plasticity of RNA infections is well documented, and we were interested in investigating if specific genetic mutations in EBOV would be observed in the ferret eye at high frequencies, suggesting specific adaptations are key to allowing persistence. One of the specific mutations was a stop codon in the L protein in approximately 16% of the genomes. This genome encodes a truncated, presumably defective viral polymerase in this tissue and may result in a lower viral load. Interestingly, stop codons in the L protein were also identified in humans with EVD at the minor variant level, and these correlated with lower viral loads (28). If high viral loads are associated with recrudescence, then lower viral loads may be important for persistence, and a defective polymerase would provide a mechanism to explain persistence and support a model in which genomes are replicated at lower frequencies (28). Another explanation is that functional, highly infective EBOV genomes are not required for persistent virus in the eyes, since the observation that little genetic drift appears to occur in persistent compartmentalized virus implies viral persistence is maintained due to stable nonreplicating virus invisible to the immune system and not periodic reinfections to maintain titer. Isolation and characterization of virus from the eyes of infected ferrets may provide an avenue for investigation of this phenomenon.

These results further support the development of the ferret model to determine its application in the evaluation of EVD MCMs. Although we expect the cynomolgus monkey to remain the critical model for demonstration of efficacy and determination of dose and regimen under the FDA Animal Rule, a small-animal model that recapitulates some aspects of EBOV infection would provide an affordable model for MCM screening and hypothesis testing with regard to EBOV.

## MATERIALS AND METHODS

### Viruses and cells.

Zaire ebolavirus (strain Kikwit-95, EBOV-Kikwit-95) HCM/SAV/43, produced and characterized by HCM PHE Porton Down, was sourced from BEI Resources with support from NIAID. The virus was passaged to P3 in Vero E6 African green monkey kidney cells (Vero C1008; NR-596; BEI Resources). The titer was determined as 5.6 × 10^6^ median tissue culture infectious dose (TCID_50_)/1.35 × 10^4^ PFU/ml. The virus stock was sterile and absent of mycoplasma, and the endotoxin level was <1.0 EU/ml. Virus morphology was confirmed by electron microscopy. Viral RNA was detected and quantified at 4.87 × 10^11^ genome equivalents/ml by a Zaire specific real-time PCR (29), and the stock was deep sequenced, revealing 86.6% of 7-uridine virus was produced.

### Animals.

Thirty-three healthy ferrets (Mustela putorius furo) aged between 8 and 10 weeks old were obtained from a UK Home Office accredited supplier (Highgate Farm, UK). The mean weight at the time of challenge was 505 g/ferret (range, 407 to 647 g). Animals were housed in groups of 3 at Advisory Committee on Dangerous Pathogens (ACDP) containment level 4. Cages met with the UK Home Office Code of Practice for the Housing and Care of Animals Bred, Supplied or Used for Scientific Procedures (December 2014). Access to food and water was *ad libitum*, and environmental enrichment was provided. All experimental work was conducted under the authority of a UK Home Office-approved project license that had been subject to local ethical review at PHE Porton Down by the Animal Welfare and Ethical Review Body (AWERB) as required by the Home Office Animals (Scientific Procedures) Act 1986.

### Experimental design.

Before the start of each experiment, animals were randomly assigned to challenge groups to eliminate bias. The weight distribution of the animals was tested to ensure there was no statistically significant difference between groups (one-way analysis of variance, *P* > 0.05). An identifier chip (Bio-Thermo Identichip, Animalcare Ltd., UK) was inserted subcutaneously into the dorsal cervical region of each animal. Prior to challenge, animals were sedated by inhalation anesthetic (isoflurane). The virus (100 μl) was injected in the right cranial thigh muscle.

Four dose-ranging experiments were performed. In the first study, 6 female ferrets were split into 2 groups (*n* = 3), and each group was infected with 5.6 × 10^4^ TCID_50_ or 5.6 × 10^1^ TCID_50_ of EBOV-Kikwit-95. Animals were scheduled for euthanasia on day 6 postchallenge. In the second study, 9 female ferrets were split into 3 groups (*n* = 3) and infected with 5.6 × 10^1^, 5.6 × 10^0^, or 5.6 × 10^−1^ TCID_50_ of EBOV-Kikwit-95. Animals were scheduled for euthanasia on day 14. In the third and fourth dose ranging studies, 9 female or 9 male ferrets were split into 3 groups (*n* = 3) and infected with a high (5.6 × 10^−1^ TCID_50_), medium (5.6 × 10^−2^ TCID_50_), or low (5.6 × 10^−3^ TCID_50_) dose of EBOV-Kikwit-95.

Whole blood, oral swabs, and rectal swabs were taken once prechallenge and then daily postchallenge for at least one ferret per dose group. At necropsy, whole blood, oral swabs, and rectal swabs were taken alongside tissue samples for histopathology. Tissue samples (lung, liver, kidney, spleen, reproductive organs [ovaries/testes], and eyes) were also collected and added to RNAlater stabilization solution to preserve the RNA transcriptome and stored at –80°C until assay.

### Clinical and postmortem observations.

Animals were monitored for clinical signs of disease twice daily until a critical phase of the study was reached, and then the frequency of monitoring was increased to 4 to 6 times per day (approximately 4 to 6 h apart). Clinical signs of disease were assigned a score based upon the following criteria: healthy, 0; arched back, 1; dehydrated/not drinking, 1; rash, 1; gait changes, 1; wasp waisted, 1; ruffled fur, 1; nasal discharge, 1; sneezing, 1; shivering, 1; lethargic, 2; vomiting, 2; depression, 2; diarrhea, 2; labored breathing, 3; temperature >39°C, 1; temperature >40°C, 2; weight loss, >5% from maximum, 1; weight loss, >10% from maximum, 2.

The following were criteria for immediate euthanasia: immobility defined as a lack of movement even after stimulus, such as handling, clinical score of 9; neurological signs including repetitive or unusual movement or disorientation, clinical score of 9; a sudden drop in temperature greater than 3°C in less than 24 h; and weight loss at or above 30% of baseline weight for two consecutive scheduled readings within 24 h. Presentation of multiple clinical symptoms incompatible with survival were also grounds for euthanasia.

Temperature was taken using a microchip reader and implanted temperature/ID chip (Animalcare Bio-Thermo Identichip). Temperature was recorded at each clinical scoring point using the chip to ensure any peak of fever was recorded. Animals were weighed at the same time of each day from the day before infection until euthanasia.

### Necropsy procedures.

Exsanguination was performed on anesthetized ferrets via cardiac puncture and blood volume removal, followed by injection of an anesthetic overdose (140 mg/kg of body weight sodium pentobarbital; Dolelethal; Vetquinol UK Ltd.). A necropsy was performed immediately after confirmation of death. At necropsy, blood samples were collected into lithium heparin and processed within 1 h using a VetScan VS2 chemistry analyzer to obtain blood chemistry data with the comprehensive diagnostic panel rotor. Blood, oral, and rectal swab fluids were collected into AVL buffer, ethanol was added, and samples were stored below –60°C. Sections of lung, liver, spleen, kidney, reproductive tract, and whole eye were collected into neutral buffered formalin (NBF) for histology and RNAlater for extraction and analysis by RT-qPCR.

### RNA extraction.

Total RNA was extracted from blood, oral, and rectal fluids using the QIAamp viral RNA kit (Qiagen) by following the manufacturer’s instructions and eluted in 60 μl AVE buffer. Total RNA was purified from tissues using the RNeasy minikit (Qiagen). One piece of RNAlater-stabilized tissue was disrupted in Qiagen buffer RLT containing beta-mercaptoethanol and homogenized using the Bertin Minilys homogenizer. A volume of homogenate equal to 30 μg of tissue was added to additional RLT buffer to give a final volume of 600 μl, and the lysates were stored below –60°C. The lysates were subsequently thawed, and an equal volume of 600 μl 70% ethanol was added. Samples were extracted using the RNeasy minikit by following the manufacturer’s instructions and eluted in 90 μl RNase-free water.

### Quantification of viral loads by RT-qPCR.

The RT-qPCR was based on a species-specific filovirus RT-PCR (29). This assay is specific for the EBOV nucleoprotein (NP) region and the amplicon size is 76 bp, nucleotides (nt) 518 to 593 in Mayinga Zaire 1976 AF086833.2, using primers F565 (5′-TCT GAC ATG GAT TAC CAC AAG ATC-3′) and R640 (5′-GGA TGA CTC TTT GCC GAA CAA TC-3′) and probe p597s (6FAM-AGG TCT GTC CGT TCA A-MGBNFQ). The 95% lower limit of detection (LLOD_95_) was determined during assay development and was calculated as 473 genome copies/ml sample (5.5 genome copies/reaction). The lower limit of quantification (LLOQ) of the RT-qPCR is 4,300 genome copies/ml sample (50 genome copies/reaction). For statistical and graphical purposes, undetermined samples or samples determined as less than the LLOD_95_ have been assigned a value equivalent to the theoretical minimum detectable amount (TMDA) (86 genome copies/ml).

For tissue samples, the TMDA of the RT-qPCR is 0.6 genome copies/mg tissue (1 genome copy/reaction), the LLOD_95_ is 3.3 genome copies/mg tissue, and the LLOQ is 30 genome copies/mg tissue (50 genome copies/reaction). For statistical and graphical purposes, undetermined samples or samples determined as less than the LLOD_95_ have been assigned a value equivalent to the TMDA (0.6 genome copies/mg).

### Histopathological analysis.

Samples from spleen, kidney, lung, reproductive tract (including uterus and ovaries for females and testis and epididymis for males), eye globe, including appendages, and liver from each animal were fixed in 10% neutral buffered formalin and processed to paraffin wax, and 3- to 5-μm thick sections were cut and stained with hematoxylin and eosin (H&E). The stained tissue sections were scanned using a 3D-Histech panoramic slide scanner, examined by using Caseviewer software v2.3, and evaluated subjectively. Slides were randomized prior to examination to remove prior knowledge of group or treatment to prevent bias (blind evaluation).

For each organ, different parameters (lesion profiles) were evaluated and scored from 0 to 4: 0, within normal limits; 1, minimal; 2, mild; 3, moderate; and 4, severe.

The selected parameters to evaluate the histopathology were (i) spleen, congestion and/or hemorrhages, necrosis, presence of polymorphonuclear leukocytes (PMNs) and lymphoid depletion (observed in the splenic white pulp); (ii) liver, congestion and/or hemorrhages, hepatocellular fat degeneration, presence of viral inclusion bodies, and necrosis; (iii) kidney, congestion and/or hemorrhages and necrosis; (iv) lung, congestion and/or hemorrhages and necrosis; (v) reproductive tract, congestion and/or hemorrhages and necrosis; and0 (vi) eye, presence of any lesion evaluated.

### Presence of virus RNA by *in situ* hybridization.

Consecutive tissue sections were stained by an RNAScope *in situ* hybridization (ISH) technique to detect viral RNA. RNA ISH was performed with an RNAscope 2.5 HD detection kit (Advanced Cell Diagnostics, Bio-Techne) according to the manufacturer’s instructions. In brief, sections were incubated for 1 h at 60°C and deparaffinized in xylene and 100% alcohol. Hydrogen peroxide treatment for 10 min at room temperature quenched endogenous peroxidases. Slides were incubated for 15 min in boiling RNAscope target retrieval reagents and incubated at 40°C for 30 min in RNAscope protease plus before a commercial specific Ebola probe (V-ZaireEbola-NP-sense probe 448581; ACD Bio-Techne) was incubated for 1 h at 40°C before hybridization. RNA was visualized using Fast Red chromogenic stain. Tissues were counterstained with Gill’s no. 1 hematoxylin. Slides were scanned and evaluated subjectively and quantitatively for presence of virus RNA in the sections. The presence of EBOV nucleic acid in spleen, liver, lung, kidney, and reproductive tract organs was evaluated and quantified using NIKON NIS-Ar Advanced Research Imaging Software, calculating the area of tissue positive for RNAScope staining. For the eye sections, a semiquantitative analysis was used to evaluate the presence of virus RNA within the different structures: cornea, lens, vitreous, aqueous humor, retina, choroid, sclera, iris, ciliary bodies, optic nerve, conjunctiva, and adnexal glands. The scoring system used was the following: −, no presence of viral RNA; −/+, inconclusive presence of small amount of viral RNA; +, moderate presence of viral RNA; +++, abundant presence of viral RNA.

### RNA sequencing.

For sequencing of the lung, liver, spleen, kidney, and ovary samples on day 5 and day 6 postchallenge samples, RNA-seq libraries were prepared from the DNase-treated total RNA using the Epicenter ScriptSeq v2 RNA-Seq library preparation kit, followed by 10 to 15 cycles of amplification and purification using AMPure XP beads. Samples from 86.46% of the fatalities and 52.63% of the survivors were amplified using 15 cycles. Each library was quantified using Qubit and the size distribution assessed using the Agilent 2100 Bioanalyzer, and the final libraries were pooled in equimolar ratios. The raw fastq files generated by HiSeq2500 were trimmed to remove Illumina adapter sequences using Cutadapt v1.2.1 (30). The option “−O 3” was set so that 3′ end of any reads that matched the adapter sequence with greater than 3 bp was trimmed off. The reads were further trimmed to remove low-quality bases using Sickle v1.200 (31) with a minimum window quality score of 20. After trimming, reads shorter than 10 bp were removed.

For sequencing of the testes samples on day 5 and day 6 postchallenge, RNA-seq libraries for amplicons generated by ARTIC were prepared following the PCR tiling of Ebola virus with native barcoding protocol provided by Oxford Nanopore Technologies using LSK109 and EXP-NBD104/114. Amplicons generated by ARTIC PCR were purified and normalized to 200 fmol before DNA end preparation and barcode and adapter ligation. The library was loaded onto a FLO-MIN106 flow cell, and sequencing reads were called with Guppy using the high-accuracy calling parameters.

### Consensus virus genomes.

For the lung, liver, spleen, kidney, and ovary samples, Hisat2 v2.1.0 (32) was used to map the trimmed reads on the *Mustela putorius* reference genome assembly (release-91) downloaded from the Ensembl FTP site. The unmapped reads were extracted by bam2fastq (v1.1.0) and then mapped on a known EBOV genome (GenBank sequence accession no. KY426690) using Bowtie2 v2.3.5.1 (32) by setting the options to parameters “–local −X 2000 –no-mixed,” followed by sam file to bam file conversion, sorting, and removal of the reads with a mapping quality score below 11 using SAMtools v1.9 (33). After that, the PCR and optical duplicate reads in the bam files were discarded using MarkDuplicates in the Picard toolkit v2.18.25 (http://broadinstitute.github.io/picard/) with the option “REMOVE_DUPLICATES=true.” The resultant bam file was processed by Quasirecomb v1.2 (34) to generate a Phred-weighted table of nucleotide frequencies that were parsed with a custom Perl script to generate a dominant genome sequence, as in our previous description (35). The dominant genome sequence was then used as a template in the second round of mapping to generate a reference genome sequence.

For the testes samples, the artic-ncov2019 pipeline v1.2.1 (https://artic.network/ncov-2019/ncov2019-bioinformatics-sop.html) was used to filter the parsed fastq files produced by Nanopore sequencing with lengths between 400 and 700 bp. This pipeline then processed the filtered reads and corresponding fast5 files to generate primer-clipped bam files and consensus genome sequences.

### Minor variations of amino acids in the EBOV proteome.

For the lung, liver, spleen, kidney, and ovary samples, the minor variations of amino acid in the genes of virus were called, as in our previous description (28). Reads (unmapped on ferret genome) were aligned to the reference EBOV dominant genome sequence using Bowtie2 with the parameter of “–local −X 2000 –no-mixed.” The Bowtie2 outputs were processed in the same way to generate a bam file without read duplication. This bam file was then processed by diversiutils script in DiversiTools (http://josephhughes.github.io/btctools/) with the “-orfs” function to generate the number of amino acid changes caused by the nucleotide deviation at each site in the protein. To distinguish low-frequency variants from Illumina sequence errors, diversiutils used the calling algorithms based on the Illumina quality scores to calculate a *P* value for each variant at each nucleotide site (36). The amino acid change was then filtered based on the *P* value (<0.05) to remove the low-frequency variants from Illumina sequence errors, and only the sites with amino acid coverage of >5 were taken into account.

For the testes samples, the bam files generated by the artic-ncov2019 pipeline were then processed by diversiutils script in DiversiTools (http://josephhughes.github.io/btctools/) with the “-orfs” function to generate the number of amino acid changes caused by the nucleotide deviation at each site in the protein. Only sites with amino acid coverage of >10 were taken into account.

### Data availability.

All data and materials used in the analysis are presented in the main text and supplemental material.
