# Supplementary material for: Dose-Dependent Response to Infection with Ebola Virus in the Ferret Model and Evidence of Viral Evolution in the Eye
Source: J Virol. 2021 Nov 23;95(24):e00833-21. doi: 10.1128/JVI.00833-21 (PMC8610581; doi:10.1128/JVI.00833-21)
Supplement: Supplementary file 1 — Tables S1 to S4; Fig. S1 to S5. Download jvi.00833-21-s0001.pdf, PDF file, 1.5 MB [file jvi.00833-21-s0001.pdf]

Supplementary Table 1. Characterisation of Zaire Ebolavirus kikwit	
Sequence analysis of Glycoprotein gene	100% homology with Mayinga EBOV Glycoprotein gene
Deep sequence analysis	86.8 % 7U
Plaque assay titre	1.35 X 10^4 pfu / mL
$TCID_{50}$ titre	5.6 X 10^6 $TCID_{50}$ / mL
Genomic RNA qRT-PCR	4.87 X 10^{11} GE / mL
Virus particle count TEM[1]	2.2 X 10^9 Vp / mL
Virus particle count ViroCyt[2]	5.5 X 10^8 Vp / mL
Ratio of genomic equivalents to PFU	36074074:1
Ratio of virus particles (Virocyt) to PFU	40740:1

Supplementary Table 1. Zaire ebolavirus (Strain Kikwit-95) characterisation. The Zaire ebolavirus stock used for these experiments has been extensively characterised. The stock has been sequenced, titred and particle ratios have been calculated. [1]By Transmission Electron Microscope Particle Count. [2]As determined using the ViroCyt 3100 Virus counter.

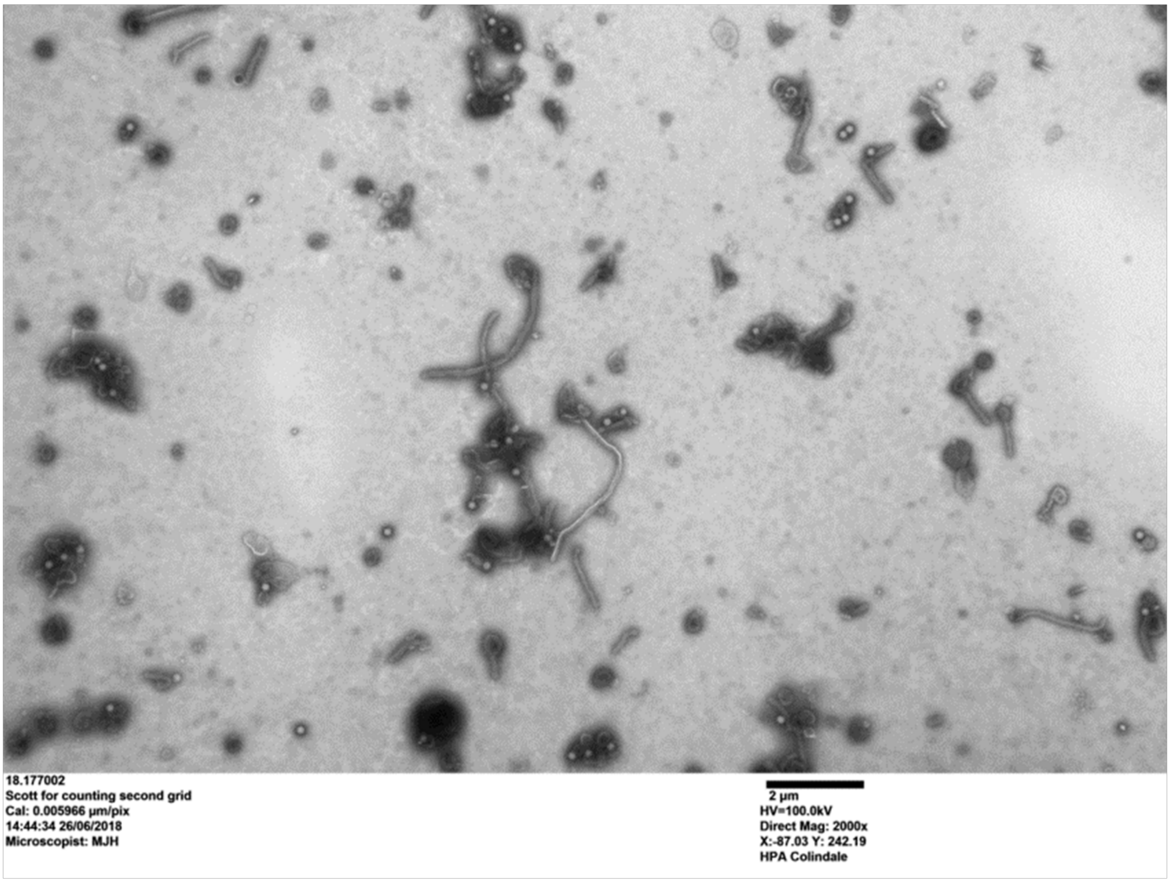

Supplementary Fig. 1 Electron Microscopy of Zaire ebolavirus (Kikwit-95) stock. The Ebola virus stock was observed under electron microscopy. Typical filovirus particles can be observed in the centre of the image and smaller particles can be observed on the outer edges of the image.

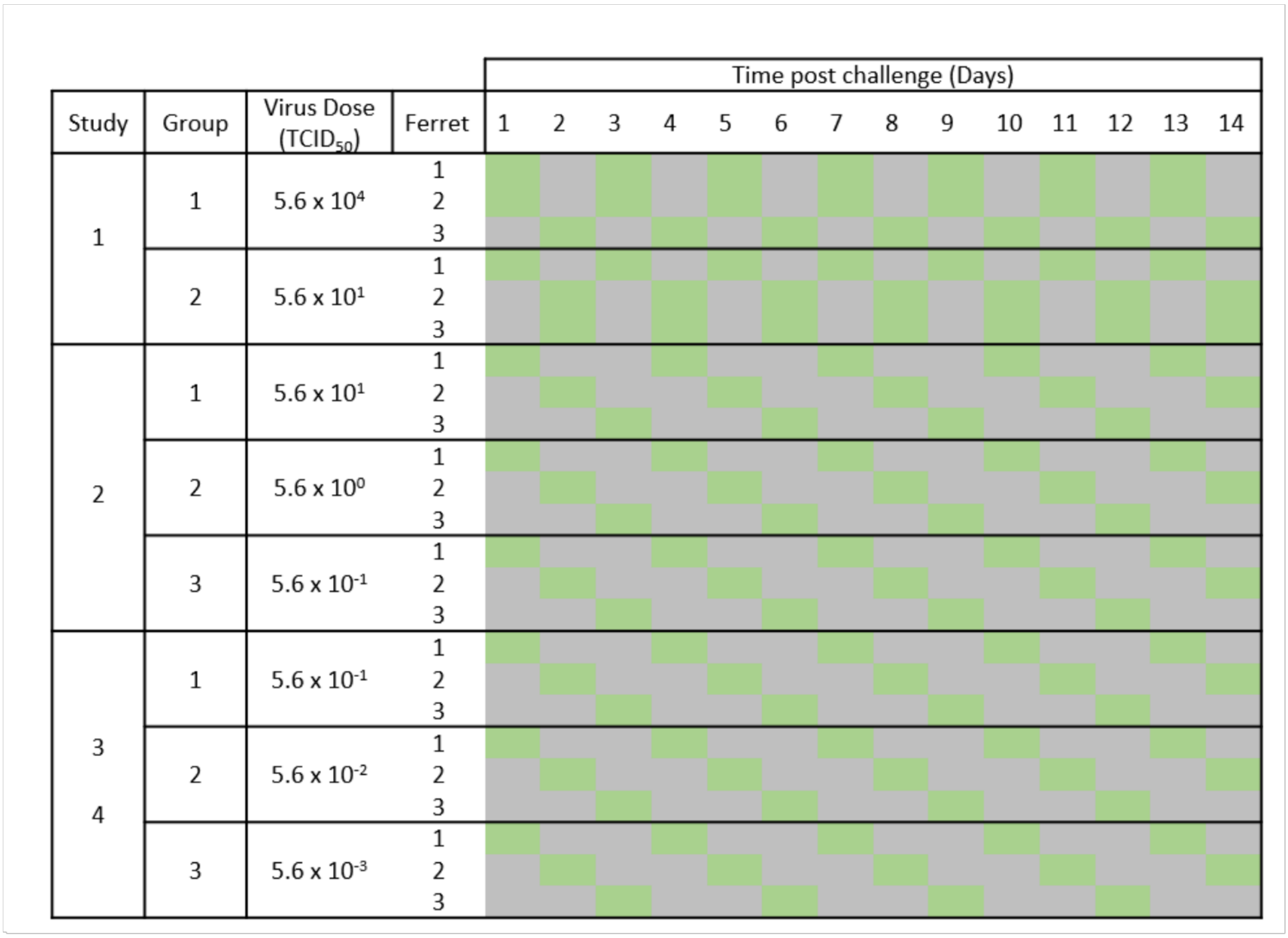

Supplementary Fig 2. Study Design. Ferrets were monitored for clinical signs, weight and temperature and were sampled daily for EDTA blood for PCR, Lithium heparin blood for VetScan blood chemistry, Oral and Rectal swabs for PCR according to the schedule.

Study	Group	Virus Dose ($TCID_{50}$)	Animal ID	Time to euthanasia (day)	Clinical signs at euthanasia decision	Total summed clinical score
1	1	5.6 x 10^4	18011	3.26	G, A, Lb	5
			18453	2.76	G, A, L	6
			18568	3.26	G, A	2
	2	5.6 x 10^1	17193	4.09	W, G, A	3
			18016	5.09	W, G, A, Lb, S, D	10
2	1	5.6 x 10^1	00088	5.03	W, A, R, D, L, V, Do	20
			16385	5.03	W, A, Lb, R, Ra, D, L, V, Do	23
			00468	4.17	W, G, A, Lb, L	10
	2	5.6 x 10^0	99894	5.44	W, G, A, Lb, R, L	10
			00545	5.37	W, G, A, Lb, R, Ra, L	11
			00230	5.37	W, G, A, Lb, R, Ra, L	13
	3	5.6 x 10^{-1}	21954	5.84	A, Lb, R, Ra, L	9
			01755	5.84	W, A, R, Ra, L	8
			99938	5.84	W, A, Lb, R, I	17
3	1	5.6 x 10^{-1}	23347	5.25	W, G, A, R, L	9
			21040	5.43	W, G, Lb, R, L, De, S	15
			23085	6.18	G, A, R, L, ND	10
	2	5.6 x 10^{-2}	23294	5.19	W, G, A, R, L, *	8
			21413	5.16	W, G, Lb, R, L	11
			21378	13.89	H	1
	3	5.6 x 10^{-3}	22944	13.89	H	0
			21518	13.89	H	0
			21428	13.89	H	0
4	1	5.6 x 10^{-1}	20136	5.06	L, W, A, G, R, Lb, D	12
			15859	5.06	L, W, A, G, R, Lb, D, Sh	13
			20016	5.06	L, W, A, G, R, Lb, D	10
	2	5.6 x 10^{-2}	16307	5.87	W, R, A, G, L, D, Lb	11
			15855	5.87	A, W, G, R, D, L	9
			18068	5.06	A, G, W, L, D	8
	3	5.6 x 10^{-3}	19712	13.87	H	0
			19717	13.87	H	0
			19857	13.87	H	0

H, Healthy (0); A, Arched back (1); D, Dehydrated/Not drinking (1); Ra, Rash (1); G, Gait changes (1); W, Wasp waisted (1); R, Ruffled fur (1); ND, Nasal discharge (1); S, Sneezing (1); Sh, Shivering (1); L, Lethargic (2); V, Vomiting (2); De, Depression (2); *, Diarrhoea (2); Lb, Laboured Breathing (3); Do, Disorientated (9), I, Immobile (9).
Temperature >39°C (1); Temperature >40°C (2).
Weight loss >5% from maximum (1); Weight loss >10% from maximum (2).

Supplementary Table 2 Clinical scores. The clinical scores were calculated according to the values in parenthesis after the clinical sign. Total clinical scores include temperature and weight changes.

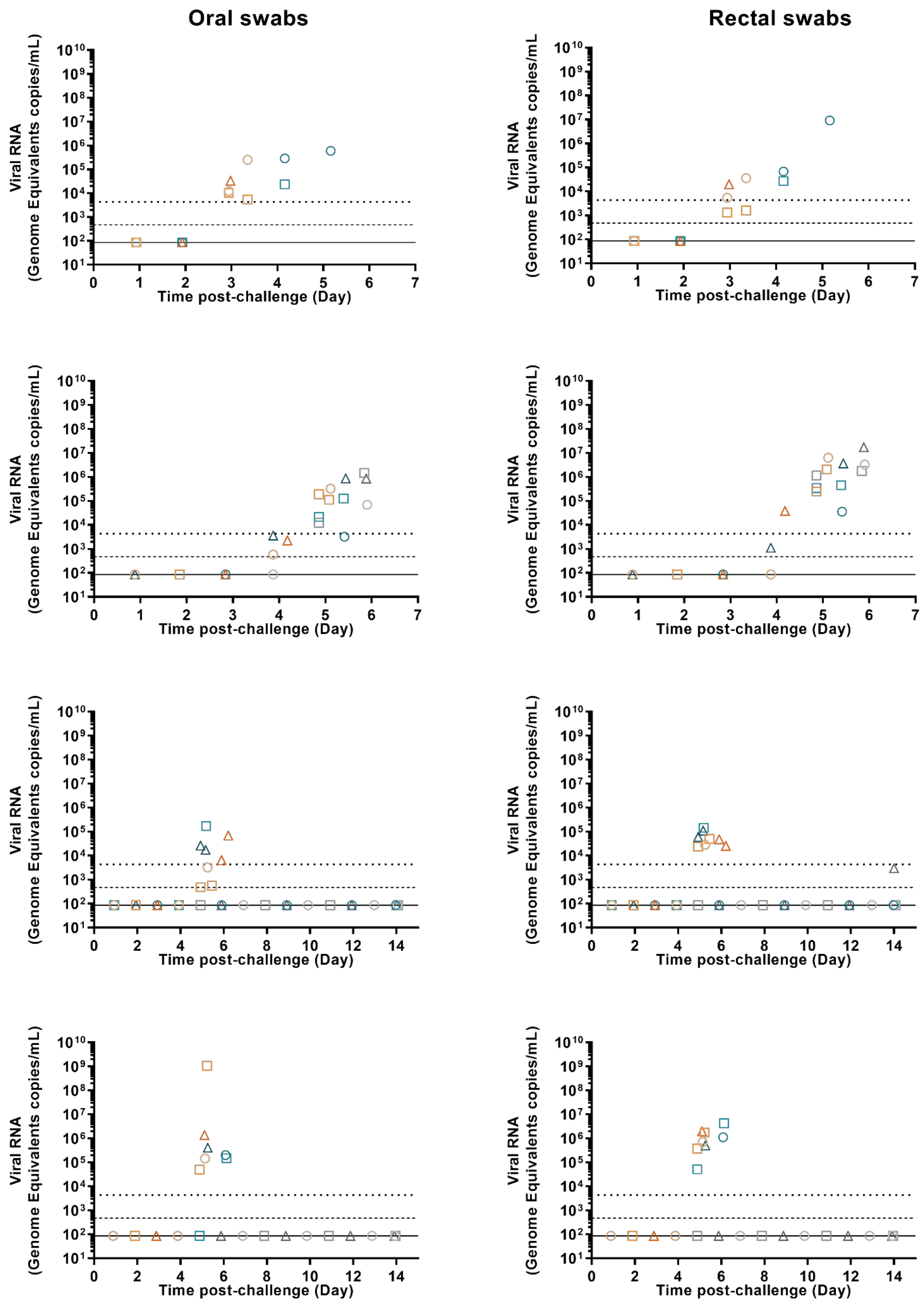

Supplementary Fig. 3 Viral load in oral and rectal samples. Viral load was quantified in oral and rectal swab samples by qRT-PCR. Group 1 5.6 x 10^{-1} $TCID_{50}$ – orange symbols, Group 2 5.6 x 10^{-2} $TCID_{50}$ teal symbols, Group 3 5.6 x 10^{-3} $TCID_{50}$ grey symbols. Upper dotted line is the lower limit of quantification (LLOQ), Dashed line is limit of detection (LOD) and solid line is the theoretical minimum detectable amount (TDMA).

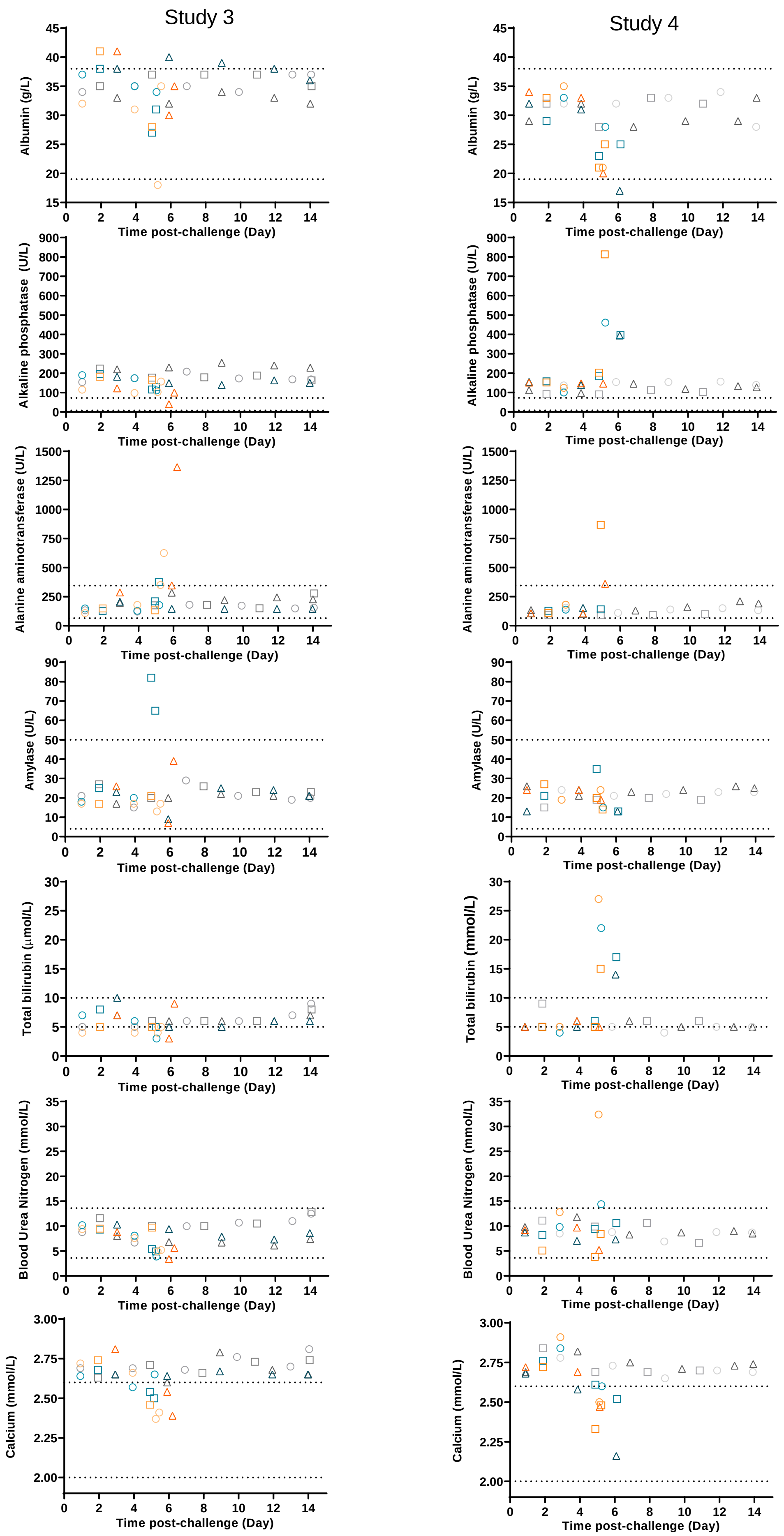

Supplementary Fig. 4 VetScan® VS2 Comprehensive panel blood chemistry (Albumin to Calcium). The parameters measured were albumin (ALB), alanine aminotransferase (ALT), alkaline phosphatase (ALP), amylase (AMY), total bilirubin (TBIL), and urea nitrogen (BUN) and total calcium (CA++) in heparinized whole blood. The upper and lower dashed lines represent the manufacturers reference range for a ferret. Group 1 5.6 x 10^{-1} $TCID_{50}$ – orange symbols, Group 2 5.6 x 10^{-2} $TCID_{50}$ teal symbols, Group 3 5.6 x 10^{-3} $TCID_{50}$ grey symbols.

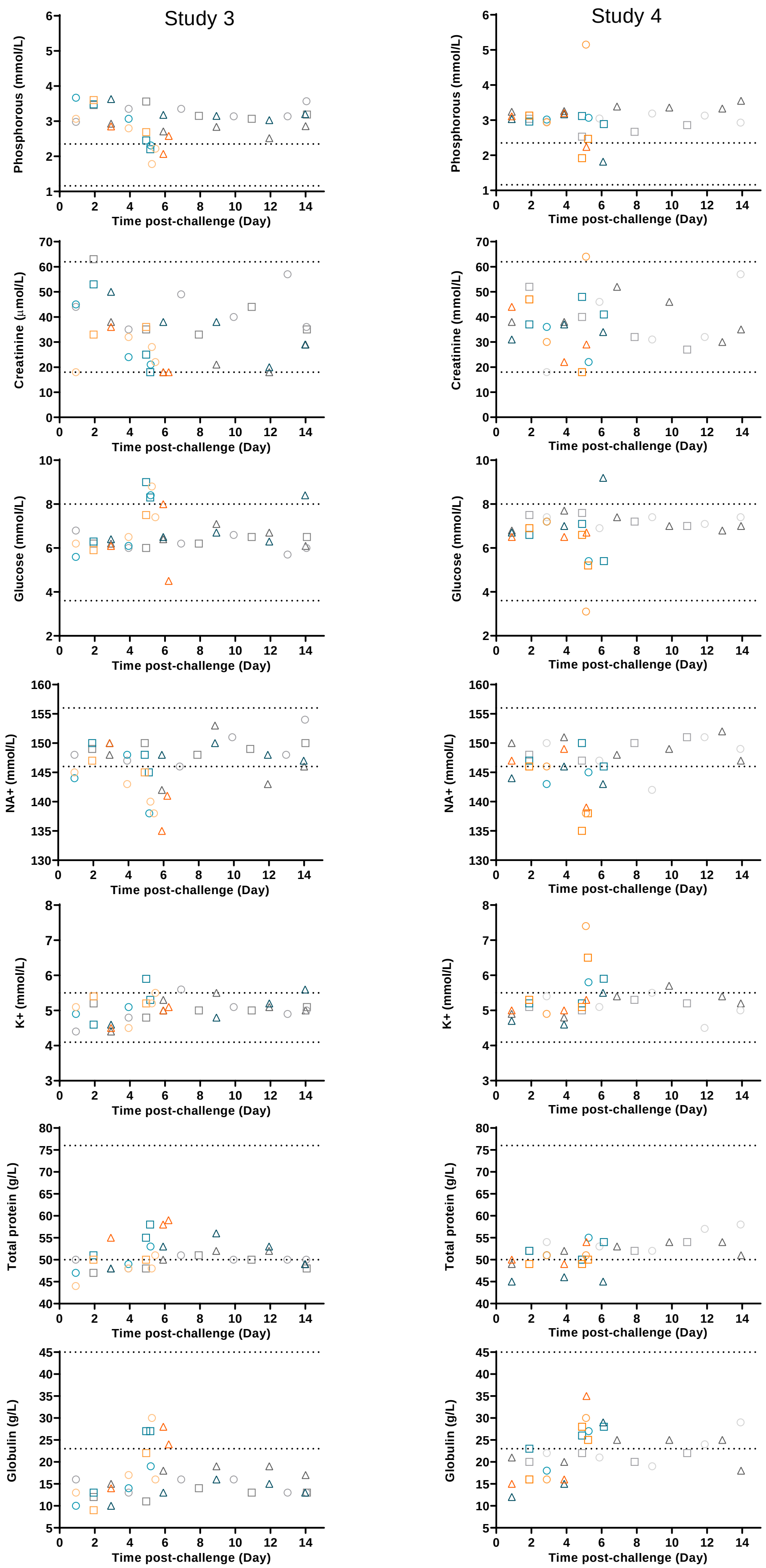

Supplementary Fig. 5 VetScan® VS2 Comprehensive panel blood chemistry (Phosphorous to Globulin). The parameters measured were phosphorus (PHOS), creatinine (CRE), glucose (GLU), sodium (NA+), potassium (K+), globulin (GLOB) and total protein (TP) in heparinized whole blood. The upper and lower dashed lines represent the manufacturers reference range for a ferret. Group 1 5.6×10^{-1} $TCID_{50}$ – orange symbols, Group 2 5.6×10^{-2} $TCID_{50}$ teal symbols, Group 3 5.6×10^{-3} $TCID_{50}$ grey symbols.

Study	Group	Virus Dose ($TCID_{50}$)	Animal	Histo ref	Spleen	Liver	Lung	Kidney	Ovary	Oviduct	Testis
1	1	5.6 x 10^4	18568	122/19	**0.9**	**0.3**	**0**	**0.1**	**0.1**	**0.02**	
1	1	5.6 x 10^4	18011	123/19							
1	1	5.6 x 10^4	18453	124/19							
1	2	5.6 x 10^1	17193	125/19	**0.7**	**1.7**	**0.2**	**0.2**	**0.3**	**0.03**	
1	2	5.6 x 10^1	18016	126/19							
1	2	5.6 x 10^1	17865	127/19							
2	1	5.6 x 10^1	00088	412/19	**0.9**	**2**	**0.12**	**0.3**	**0.1**		
2	1	5.6 x 10^1	00468	414/19							
2	1	5.6 x 10^1	16385	417/19							
2	2	5.6 x 10^0	00230	413/19	**1.5**	**0.7**	**0.2**	**0.1**		**0**	
2	2	5.6 x 10^0	00545	415/19							
2	2	5.6 x 10^0	99894	419/19							
2	3	5.6 x 10^{-1}	01755	416/19	**8.9**	**4.1**	**2.3**	**0.8**	**0.3**	**0.2**	
2	3	5.6 x 10^{-1}	21954	418/19							
2	3	5.6 x 10^{-1}	99938	420/19							
3	1	5.6 x 10^{-1}	21040	455/19	**1.54**	**0.38**	**0.02**	**0.04**	**0.02**	**0.01**	
3	1	5.6 x 10^{-1}	23085	461/19	**0.62**	**0.4**	**0.04**	**0.02**	**0.04**	**0.02**	
3	1	5.6 x 10^{-1}	23347	463/19							
3	2	5.6 x 10^{-2}	21378	456/19							
3	2	5.6 x 10^{-2}	21413	457/19	**0.8**	**0.4**	**0.07**	**0.01**	**0.01**	**0**	
3	2	5.6 x 10^{-2}	23294	462/19							
3	3	5.6 x 10^{-3}	21428	458/19							
3	3	5.6 x 10^{-3}	21518	459/19							
3	3	5.6 x 10^{-3}	22944	460/19	**0**	**0**	**0**	**0**	**0**	**0**	
4	1	5.6 x 10^{-1}	20136	642/19	**6.2**	**2.6**	**0.2**	**0.17**			**0.2**
4	1	5.6 x 10^{-1}	15859	643/19	**11.8**	**5.4**	**1.4**	**1.5**			**2.3**
4	1	5.6 x 10^{-1}	20016	644/19	**7.8**	**5.9**	**1.02**	**0.82**			**1.34**
4	2	5.6 x 10^{-2}	18068	645/19							**0.14**
4	2	5.6 x 10^{-2}	16307	646/19							
4	2	5.6 x 10^{-2}	15855	647/19	**5.7**	**5.4**	**0.8**	**0.4**			**0.9**
4	3	5.6 x 10^{-3}	19717	648/19	**0**	**0**	**0**	**0**			**0**
4	3	5.6 x 10^{-3}	19712	649/19	**0**	**0**	**0**	**0**			**0**
4	3	5.6 x 10^{-3}	19857	650/19							

Supplementary Table 3. Presence of viral RNA in tissues by RNAScope. The quantification of the presence of Zaire ebolavirus (Strain Kikwit-95) nucleic acid was calculated by the percentage of area of the whole tissue section stained positively with RNAScope. The average size of the area studied was 2-4 cm^2 . Green shading represents no RNAScope staining. Light orange <1% staining and dark orange > 1% staining.

	Group	Virus Dose ($TCID_{50}$)	Animal	Histo ref	Cornea	Lens	Vitreous	Aqueous	Retina	Choroid	Sclera	Iris	Ciliary	Optic nerve	Conjunctiva/ Glands
Study 1	1	5.6×10^{4}	18568	122/19	-	-	-	-	-	+	-	-	+	-	-
	1	5.6×10^{4}	18011	123/19	-	-	-	-	-	-	-	-	-	-	-
	1	5.6×10^{4}	18453	124/19	-	-	-	-	-	-	-	-	-	-	-
	2	5.6×10^{1}	17193	125/19	-	-	-	-	-	+	-	+	+	-	-
	2	5.6×10^{1}	18016	126/19	-	-	-	-	-	+	-	+	+	-	+
Study 2	1	5.6×10^{1}	00088	412/19	-	-	-	-	-	+	-	+	+	N/A	N/A
	1	5.6×10^{1}	00468	414/19	-	-	-	-	-	-	-	-	-	-	-
	1	5.6×10^{1}	16385	417/19	-	-	-	-	-	+	-	+	-	-	+++
	2	5.6×10^{1}	00230	413/19	-	-	-/+	-	-	+	-	-	-	-	N/A
	2	5.6×10^{0}	00545	415/19	-	-	-/+	-	-	+	-	+	+++	N/A	+++
	2	5.6×10^{0}	99894	419/19	-	-	-	-	-	+++	-	+	+++	-	N/A
	3	5.6×10^{0}	01755	416/19	-	-	-/+	-	-	+	-	+++	+++	N/A	+++
	3	5.6×10^{-1}	21954	418/19	-	-	-	-	-	+	-	+	+	+	+
	3	5.6×10^{-1}	99938	420/19	-	-	-	-	-	+	-	+	+	-	N/A
Study 3	1	5.6×10^{-1}	21040	455/19	-	-	-	-	-	+	-	+	+	N/A	-
	1	5.6×10^{-1}	23085	461/19	-	-	-	-	-	-	-	+	-	-	-
	1	5.6×10^{-1}	23347	463/19	-	-	-	-	-	-	-	-	-	N/A	N/A
	2	5.6×10^{-1}	21413	457/19	-	N/A	-	-	-	-	-	-	-	N/A	N/A
	2	5.6×10^{-2}	23294	462/19	-	-	-	-	-	-	-	-	-	-	-
	2	5.6×10^{-2}	21378	456/19	-	-	-	-	-	-	-	-	-	-	-
	3	5.6×10^{-2}	21428	458/19	-	-	-	-	-	-	-	-	-	N/A	-
	3	5.6×10^{-3}	21518	459/19	-	-	-	-	-	-	-	-	-	N/A	N/A
	3	5.6×10^{-3}	22944	460/19	-	-	-	-	-	-	-	-	-	-	-
Study 4	1	5.6×10^{-3}	20136	642/19	-	-	-	-	-	+	-	+	+	N/A	+++
	1	5.6×10^{-1}	15859	643/19	-	-	-/+	-	-	+++	-	+	+++	N/A	+++
	1	5.6×10^{-1}	20016	644/19	-	-	-/+	-	-	+++	-	+++	+++	+++	+++
	2	5.6×10^{-1}	18068	645/19	-	-	-	-	-	+++	-	+	+	N/A	+
	2	5.6×10^{-2}	16307	646/19	-	-	-	-	-	+++	-	+	+	N/A	+++
	2	5.6×10^{-2}	15855	647/19	-	-	-	-	-	+++	-	+++	+++	+++	+++
	3	5.6×10^{-2}	19717	648/19	-	-	-	-	-	-	-	-	-	-	-
	3	5.6×10^{-3}	19712	649/19	-	-	-	-	-	-	-	-	-	-	-
	3	5.6×10^{-3}	19857	650/19	-	-	-	-	-	-/+	-	-	-	-	-

Supplementary Table 4. Presence of viral RNA in eyes by RNAScope. The quantification of the presence of Zaire ebolavirus (Strain Kikwit-95) nucleic acid was evaluated as absent (-), low (+), medium (++) or high (+++). Nineteen out of 25 animals that reached their euthanasia endpoint stained positively for viral RNA in at least one eye structure.
